# Comprehensive analysis identifies IFI16 as a novel signature associated with overall survival and immune infiltration of skin cutaneous melanoma

**DOI:** 10.1186/s12935-021-02409-6

**Published:** 2021-12-20

**Authors:** Hanwen Wang, Xiaoxia Xie, Junyou Zhu, Shaohai Qi, Julin Xie

**Affiliations:** Department of Burn Surgery, The First Affiliated Hospital of Sun Yat-Sen University, Guangzhou, 510080 Guangdong People’s Republic of China

**Keywords:** Bioinformatics, Feature selection algorithm, Skin cutaneous melanoma, Prognostic signature, IFI16

## Abstract

**Background:**

Skin cutaneous melanoma (SKCM) is the most common skin tumor with high mortality. The unfavorable outcome of SKCM urges the discovery of prognostic biomarkers for accurate therapy. The present study aimed to explore novel prognosis-related signatures of SKCM and determine the significance of immune cell infiltration in this pathology.

**Methods:**

Four gene expression profiles (GSE130244, GSE3189, GSE7553 and GSE46517) of SKCM and normal skin samples were retrieved from the GEO database. Differentially expressed genes (DEGs) were then screened, and the feature genes were identified by the LASSO regression and Boruta algorithm. Survival analysis was performed to filter the potential prognostic signature, and GEPIA was used for preliminary validation. The area under the receiver operating characteristic curve (AUC) was obtained to evaluate discriminatory ability. The Gene Set Variation Analysis (GSVA) was performed, and the composition of the immune cell infiltration in SKCM was estimated using CIBERSORT. At last, paraffin-embedded specimens of primary SKCM and normal skin tissues were collected, and the signature was validated by fluorescence in situ hybridization (FISH) and immunohistochemistry (IHC).

**Results:**

Totally 823 DEGs and 16 feature genes were screened. IFI16 was identified as the signature associated with overall survival of SKCM with a great discriminatory ability (AUC > 0.9 for all datasets). GSVA noticed that IFI16 might be involved in apoptosis and ultraviolet response in SKCM, and immune cell infiltration of IFI16 was evaluated. At last, FISH and IHC both validated the differential expression of IFI16 in SKCM.

**Conclusions:**

In conclusion, our comprehensive analysis identified IFI16 as a signature associated with overall survival and immune infiltration of SKCM, which may play a critical role in the occurrence and development of SKCM.

**Supplementary Information:**

The online version contains supplementary material available at 10.1186/s12935-021-02409-6.

## Introduction

Skin cutaneous melanoma (SKCM) is one of the most common malignant skin tumors. According to the survey report from International Agency for Research on Cancer, there were 287,723 new cases of SKCM and 60,712 deaths in 2018 worldwide, attracting global attention by increasing incidence and unfavorable prognosis [1, 2]. Surgical resection is currently considered as the first choice for the treatment of primary SKCM, which can completely cure the primary tumor [3]. However, for patients with tumor metastasis, the treatment is quite limited. In the current study, Furthermore, even though human beings have made remarkable achievements in the adjuvant therapies of SKCM, there are still problems such as the toxicity of chemotherapy, drug resistance, non-significant improvement of overall survival, and expensive cost of immunotherapy and targeted therapy [3]. Therefore, the high incidence rate, poor prognosis, and limited treatment of SKCM urged us to explore more genetic research of the disease to provide potential targets for its precise treatment and promote the prognosis of patients.

With the rapid development of information technology and the maturity of high-throughput technologies, gene microarray analysis has been gradually introduced into clinical research. This method can identify the expression characteristics of specific diseases and then screen out specific genetic signatures to provide the basis for subsequent experiments and contribute to precise treatment [4]. At the same time, the wide application of artificial intelligence technology also makes the development of bioinformatics more powerful. The feature selection algorithm combines the advantages of statistics and computer science, enables researchers to build models and simplify models to reduce the dimension of massive data [5].

In recent years, growing studies have shown that immune cell infiltration plays a critical role in the occurrence and progression of cancer [6, 7]. At the same time, evidence has also recognized that the composition of immune cells in tumor microenvironment may affect the therapeutic resistance and malignancy in SKCM [8, 9]. CIBERSORT works as an analytical tool that employs RNA-seq data to evaluate the expression of immune cells and obtain different immune cell proportions, which has been widely used in the research of tumor microenvironment of various tumors such as gliblastoma [10], colorectal cancer [11], and gastric cancer [12]. Nevertheless, little research has applied CIBERSORT to probe immune cell infiltration in SKCM.

**Fig. 1:**
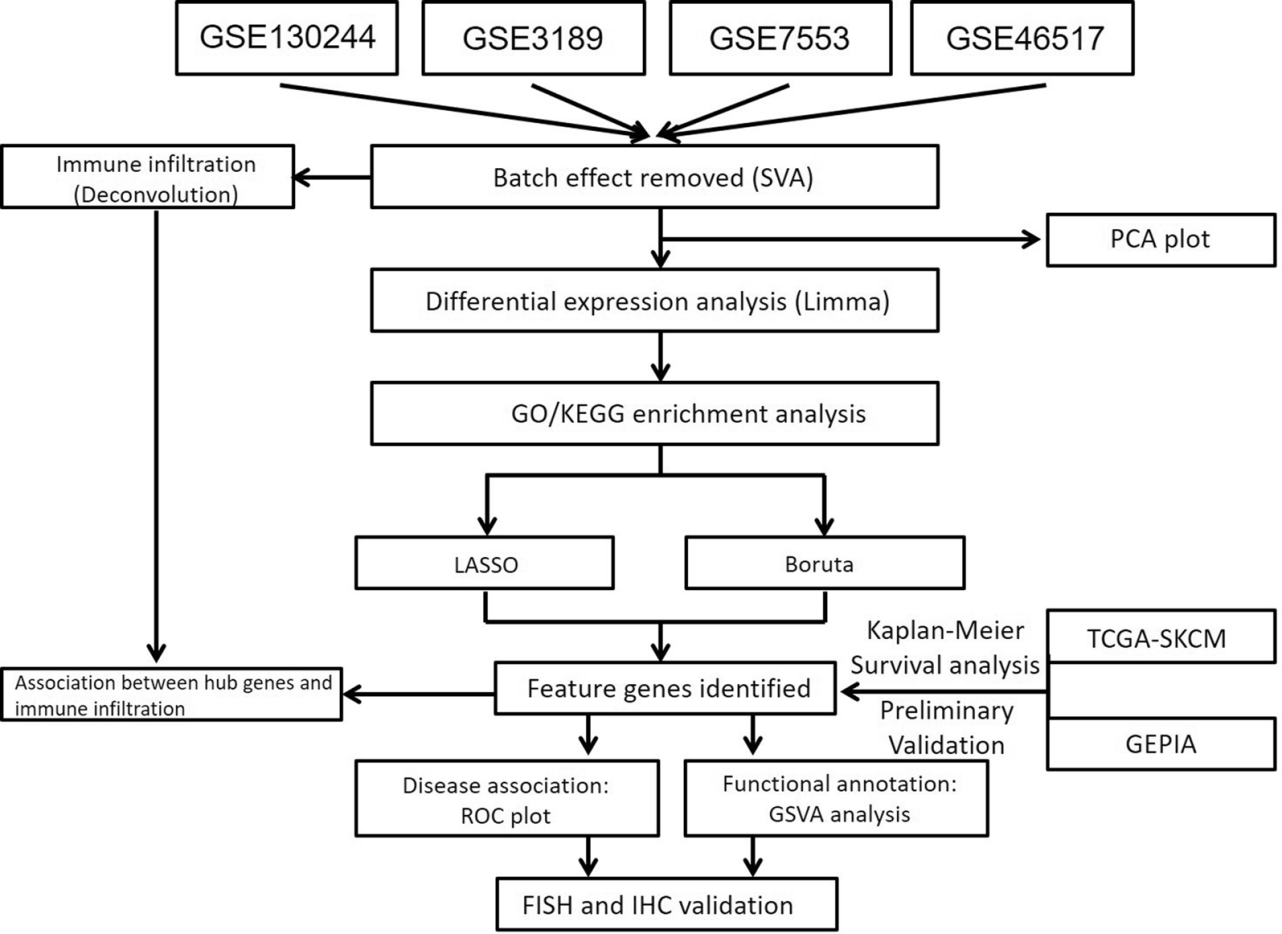
Flowchart of the research methodology

In the current study, microarray datasets of SKCM were first retrieved from the GEO and TCGA database, Differentially Expressed Genes (DEGs) between tumor and control were screened, and Gene Ontology (GO) and Kyoto Encyclopedia of Genes and Genomes (KEGG) enrichment analysis were conducted. Then, LASSO and Boruta were used to filter feature genes of SKCM, respectively. The GEPIA was applied for preliminary validation of the expression of feature genes, and then the Kaplan–Meier survival analysis was performed to screen signatures related to the overall survival of SKCM. We further analyzed the immune cell infiltration in SKCM samples by CIBERSORT and obtained the phenotype and proportion of immune cells in the tumor microenvironment. In addition, the correlation between immune cell components and the prognostic signatures was explored. Finally, paraffin-embedded specimens of SKCM and normal skin tissues were collected, and fluorescence in situ hybridization (FISH) and immunohistochemistry (IHC) were applied to validate the expression of prognostic signatures, providing the basis for research of molecular mechanism and potential approaches to novel therapeutic targets (Fig. 1).

## Materials and methods

### Microarray data mining and preprocessing

The gene expression profiles of SKCM were obtained from 4 datasets, including GSE130244, GSE3189, GSE7553 and GSE46517 of the Gene Expression Omnibus (GEO) (http://www.ncbi.nlm.nih.gov/gds/) database [13]. GSE130244 and GSE7553 were based on the GPL570 platform (Affymetrix Human Genome U133 Plus 2.0 Array), whereas GSE3189 and GSE46517 were based on the GPL96 platform (Affymetrix Human Genome U133A Array). Totally 121 samples were collected, including 99 primary SKCM samples and 22 normal skin samples. The gene expression matrices of the 4 datasets above were then combined. The inter-sample batch effect was removed using the “*sva*” R package, and the correction was visualized by two-dimensional PCA cluster plots [14].

The SKCM clinical and RNA expression data were also retrieved from the TCGA database (https://portal.gdc.cancer.gov/projects) [15]. Totally 102 primary melanoma patients from the TCGA database were enrolled in the study (Additional file 1: Table S1).

### Identification of DEGs

DEGs were identified by utilizing the *limma* R package, and a volcano map was plotted using the *ggplot2* R package to show the differential expression of DEGs [16, 17]. The identification criteria of DEGs were defined as *p* < 0.05 and |log_2_FC| > 1.

### Gene enrichment analysis

Metascape (https://metascape.org) was used to elucidate and visualize the functional enrichment of DEGs [18]. Gene Ontology (GO) functional enrichment analysis and Kyoto Encyclopedia of Genes and Genomes (KEGG) pathway enrichment analysis were performed, and the significant level was defined as *p* < 0.01.

### Feature selection by LASSO and Boruta

The least absolute shrinkage and selection operator (LASSO) logistic regression and Boruta algorithm were applied for feature selection from DEGs to key genes of SKCM [19]. LASSO regression was realized by the *glmnet* R package to reduce data dimensions, and Boruta by the *Boruta* R package to construct a random forest classifier, comparing and ranking the features by importance [20]. The overlapping genes derived from these two algorithms were regarded as feature genes.

### Preliminary validation of feature genes

Expression of selected genes in SKCM and normal skin tissues were analyzed for preliminary validation using the GEPIA online tool (http://gepia.cancer-pku.cn) [21]. The gene expression profiling included 461 SKCM samples from the TCGA database and 558 normal skin samples from the GTEx database.

### Survival analysis

To further clarify the relationship between the feature genes and SKCM prognosis, we used the *survival* R package for survival analysis [22]. Totally, 102 primary melanoma patients from the TCGA database were enrolled in the study. Subsequently, Kaplan–Meier plots were created, and log-rank *p* < 0.05 was considered statistically significant. Only the genes indicating an unfavorable or favorable prognosis in patients with SKCM were selected for further analysis.

### Model performance evaluation

Four receiver operating characteristic (ROC) curves were generated to estimate the accuracy of the screened prognostic biomarker in the original datasets. Area under curve (AUC) represents the performance of the model.

### Gene set variation analysis (GSVA)

Gene Set Variation Analysis (GSVA) is a non-parametric, unsupervised method for estimating the variation of specific gene set [23]. The corresponding gene set was downloaded from the Molecular Signature Database (http://gsea-msigdb.org), and the *GSVA* R package was used to find the differential expressed pathways and biological process between groups of high and low expression of candidate genes. In this part, *p* < 0.01 was regarded as statistically significant.

### Evaluation of immune cell infiltration

CIBERSORT is widely used to evaluate the types of immune cells in tumor microenvironment. Based on the principle of support vector regression, the expression matrix of immune cell subtypes was deconvoluted. In this study, CIBERSORT was used to analyze the data of patients with skin cutaneous melanoma to infer the relative proportion of 22 types of immune infiltrating cells, and the “*corrplot*” package was used to draw a correlation heatmap. Spearman correlation analysis was performed to recognize the relationship between the molecular signature and immune cell infiltration, and the “*ggplot2*” package was used for visualization. In this part, *p* < 0.05 was regarded as statistically significant.

### Specimen collection

In this study, 30 paraffin-embedded specimens of primary SKCM and 10 paraffin-embedded specimens of normal skin tissues were collected from the Department of Pathology, the First Affiliated Hospital of Sun Yat-sen University from January 2016 to July 2019 (Additional file 1: Table S2). The study was authorized by the Research Ethics Committee of the First Affiliated Hospital of Sun Yat-sen University. All participants have the right to know. The eligible SKCM specimens for this study had to meet the following criteria: (1) histologically confirmed as melanoma; (2) received no radiotherapy, chemotherapy or biotherapy before surgery. The exclusion criteria were as follows: (1) previous malignancies; (2) concomitant malignancies.

### FISH analysis

Briefly, SKCM and normal skin paraffin-embedded specimens were processed using the following steps: stoving, dewaxing, incubation with protease K, denaturation, probe hybridization, washing the slide, nuclear staining, and observation with confocal laser scanning microscope (Leica, Germany).

### IHC analysis

SKCM and normal skin paraffin-embedded specimens were processed using the following steps: stoving, dewaxing to water, removing melanin, antigen repair. Then, the specimens were washed with PBS three times and incubated with a IFI16 polyclonal antibody (1:100, abs169788, Abcam, UK) overnight at 4 °C. After that, specimens were washed with PBS three times and incubated with an HRP-conjugated secondary antibody (1:400, SV0001/SV0002, Boster, China) at room temperature for 1 h. At last, the slides were placed under an optical microscope (Olympus, Japan) for observation.

### Image acquisition and analysis

Adjusting to the appropriate focal length, randomly select the magnified 200 times views (eyepiece 10 times, objective 20 times) for IHC or 400 times views (eyepiece 10 times, objective 40 times) for FISH, and take photographs. The integrated optical density (IOD) and the corresponding staining area of each photograph were measured by Image Pro Plus 6.0 software. The mean optical density (MOD) was obtained by calculating the ratio, and the expression level of IFI16 gene and protein in SKCM and normal skin samples was reflected by the average value of MOD of three well stained random fields (hereinafter referred to as OD value) in each slide. GraphPad Prism 8 (GraphPad Software, USA) was used to draw figures.

### Statistic analysis

The statistical analysis was performed using R software version 3.6.1. All statistical tests are two-sided, and *p* < 0.05 is statistically significant.

## Results

### Data preprocessing and identification of DEGs

**Fig. 2:**
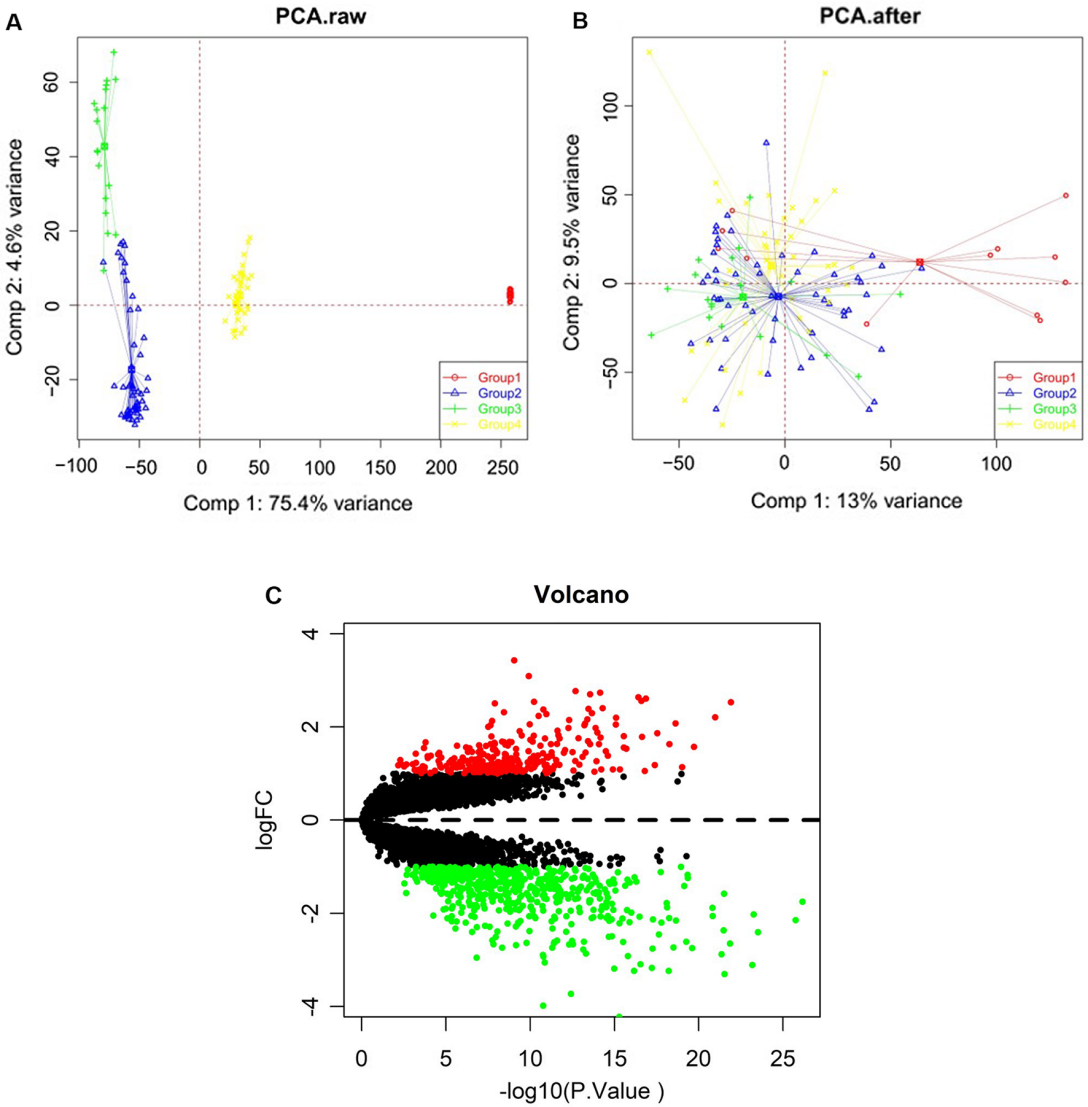
Two-dimensional PCA cluster plot before and after correction and volcano plot of DEGs. **A** Two-dimensional PCA cluster plot of GSE130244, GSE3189, GSE7553 and GSE46517 datasets before batch effect correction. **B** Two-dimensional PCA cluster plot of GSE130244, GSE3189, GSE7553 and GSE46517 datasets after batch effect correction. **C** Volcano plot of differential expressed genes (DEGs); the red represents the up-regulated genes, the black represents no significant difference genes, and the green represents the down-regulated genes

First, the batch effect was removed from the gene expression matrix after merging the GSE130244, GSE3189, GSE7553 and GSE46517 datasets, and it is presented in two-dimensional PCA cluster plots before and after preprocessing (Fig. 2A, B). The results inferred that the clustering of the four datasets was more obvious after preprocessing, indicating a more reliable data source. After data preprocessing, totally 823 DEGs were identified across all the datasets consisting of 283 up-regulated and 540 down-regulated DEGs in SKCM compared to NS samples by *limma* package of R (Fig. 2C).

### Gene enrichment analysis

**Fig. 3:**
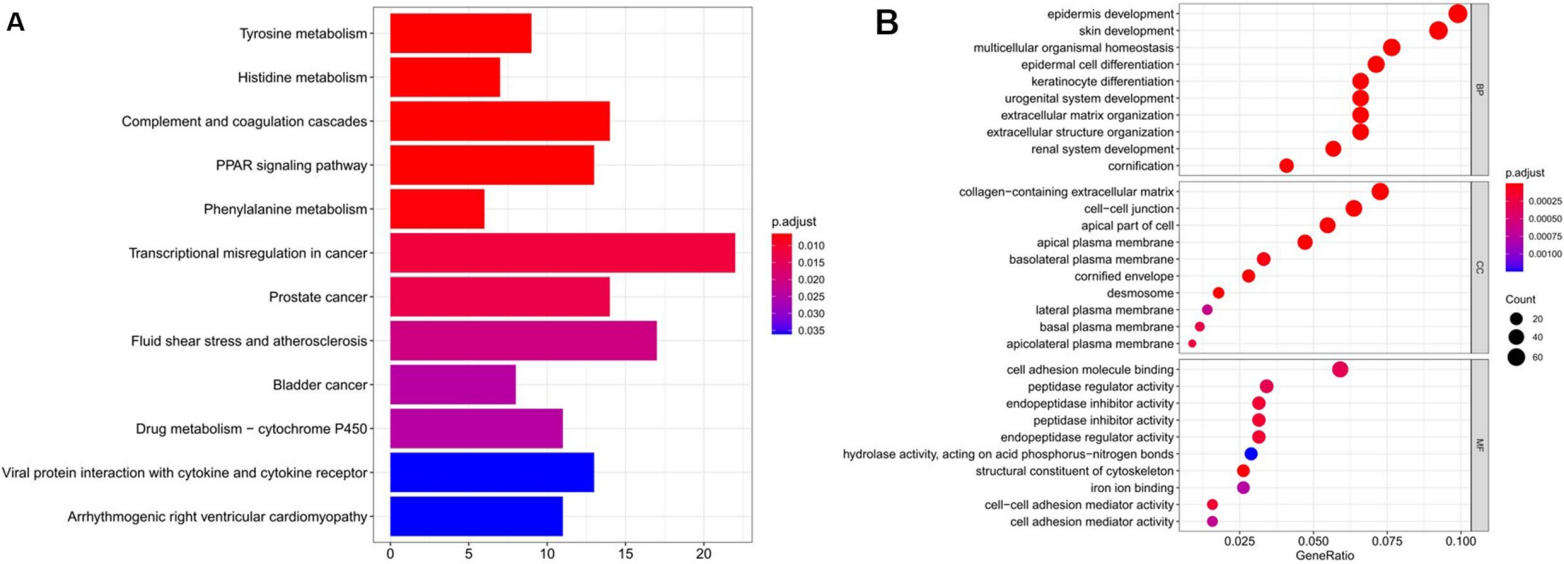
Kyoto Encyclopedia of Genes and Genomes (KEGG) pathway enrichment analysis and Gene ontology (GO) functional enrichment analysis of DEGs. **A** KEGG pathway enrichment analysis of DEGs. **B** GO functional enrichment analysis of DEGs; BP represents biological process, CC represents cell component, MF represents molecular function

Metascape was used for GO functional enrichment analysis and KEGG pathway enrichment analysis based on 823 DEGs. KEGG analysis was performed to investigate the role of these genes in various biological pathways, and we got 12 entries which were shown in Table 1 and Fig. 3A, including tyrosine metabolism (hsa00350), phenylalanine metabolism (hsa00360), transcriptional mis-regulation in cancer (hsa05202), bladder cancer (hsa05219), prostate cancer (hsa05215), etc. The results of GO enrichment analysis can classify and annotate genes through three aspects: biological process (BP), molecular function (MF) and cell component (CC), which were obtained and shown in Additional file 1: Table S3, and the top 10 entries were shown in Table 2 and Fig. 3B. The results showed that the DEGs were mostly enriched in biological processes such as epidermis development (GO:0008544), skin development (GO:0043588), cornification (GO:0070268), keratinocyte differentiation (GO:0030216), epidermal cell differentiation (GO:0009913). The above results suggested that the development and differentiation of skin tissue were playing an essential role in SKCM.

**Table 1:** Kyoto encyclopedia of genes and genomes pathway enrichment analysis of differentially expressed genes associated with SKCM

Pathway	ID	*p*-value	Count
Tyrosine metabolism	hsa00350	5.23E−05	9
Histidine metabolism	hsa00340	6.78E−05	7
Complement and coagulation cascades	hsa04610	7.52E−05	14
PPAR signaling pathway	hsa03320	9.07E−05	13
Phenylalanine metabolism	hsa00360	0.000119	6
Transcriptional misregulation in cancer	hsa05202	0.000239	22
Prostate cancer	hsa05215	0.000319	14
Fluid shear stress and atherosclerosis	hsa05418	0.000569	17
Bladder cancer	hsa05219	0.000838	8
Drug metabolism—cytochrome P450	hsa00982	0.00085	11
Viral protein interaction with cytokine and cytokine receptor	hsa04061	0.001413	13
Arrhythmogenic right ventricular cardiomyopathy	hsa05412	0.0015	11

**Table 2:** Top 10 gene ontology functional enrichment analysis of differentially expressed genes associated with SKCM

Ontology	ID	Description	*p*-value	Count
BP	GO:0008544	Epidermis development	4.08E−25	75
BP	GO:0043588	Skin development	2.35E−24	70
BP	GO:0070268	Cornification	7.36E−18	31
BP	GO:0030216	Keratinocyte differentiation	2.48E−17	50
BP	GO:0009913	Epidermal cell differentiation	5.57E−17	54
BP	GO:0001655	Urogenital system development	6.88E−16	50
BP	GO:0030198	Extracellular matrix organization	5.69E−14	50
BP	GO:0043062	Extracellular structure organization	6.33E−14	50
BP	GO:0048871	Multicellular organismal homeostasis	1.63E−13	58
BP	GO:0072001	Renal system development	2.40E−13	43
CC	GO:0062023	Collagen-containing extracellular matrix	9.17E−17	57
CC	GO:0001533	Cornified envelope	2.86E−15	22
CC	GO:0030057	Desmosome	6.66E−14	14
CC	GO:0005911	Cell–cell junction	3.21E−12	50
CC	GO:0045177	Apical part of cell	1.02E−09	43
CC	GO:0016324	Apical plasma membrane	5.41E−09	37
CC	GO:0016323	Basolateral plasma membrane	5.73E−07	26
CC	GO:0016327	Apicolateral plasma membrane	3.34E−06	7
CC	GO:0009925	Basal plasma membrane	5.14E−06	9
CC	GO:0016328	Lateral plasma membrane	1.28E−05	11
MF	GO:0005200	Structural constituent of cytoskeleton	1.11E−08	20
MF	GO:0004866	Endopeptidase inhibitor activity	5.13E−07	24
MF	GO:0098632	Cell–cell adhesion mediator activity	1.00E−06	12
MF	GO:0030414	Peptidase inhibitor activity	1.06E−06	24
MF	GO:0061135	Endopeptidase regulator activity	1.06E−06	24
MF	GO:0050839	Cell adhesion molecule binding	2.42E−06	45
MF	GO:0061134	Peptidase regulator activity	2.86E−06	26
MF	GO:0098631	Cell adhesion mediator activity	6.47E−06	12
MF	GO:0005506	Iron ion binding	8.52E−06	20
MF	GO:0016825	Hydrolase activity, acting on acid phosphorus-nitrogen bonds	1.73E−05	22

### Screening and preliminary validation of feature genes

**Fig. 4:**
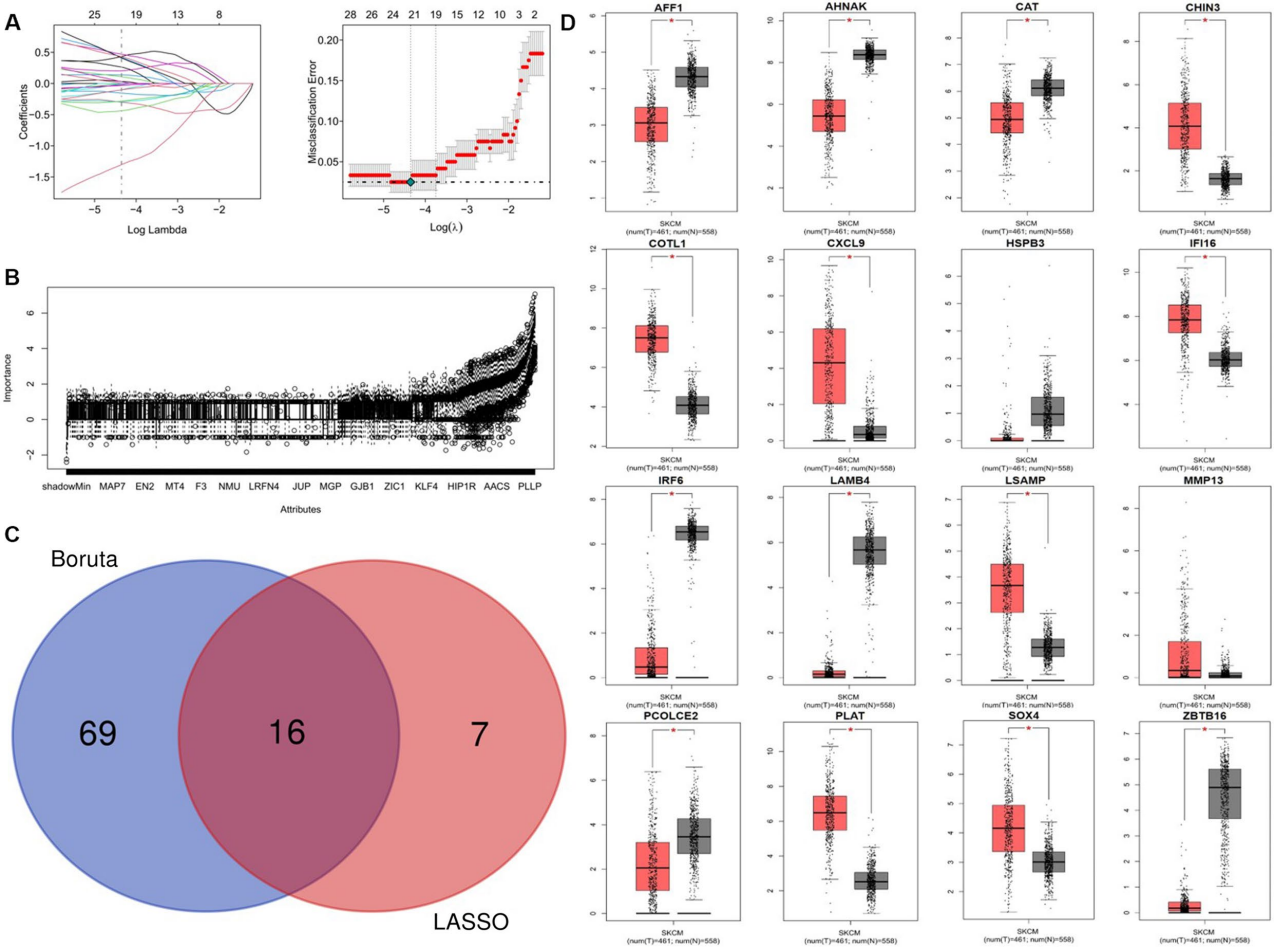
Screening and preliminary validation of feature genes. **A** Feature selection via the least absolute shrinkage and selection operator (LASSO) logistic regression model. **B** Feature selection via Boruta algorithm. **C** Venn diagram demonstrating the intersection of feature genes obtained by the two algorithms. **D** Preliminary validation of mRNA expression of intersected feature genes using the data from TCGA and GTEx databases

LASSO logistic regression algorithm was used to identify 23 feature genes from DEGs (Fig. 4A). At the same time, 85 feature genes were screened from DEGs by the Boruta algorithm (Fig. 4B). By overlapping the feature genes, we finally obtained 16 feature genes (Fig. 4C) with the best classification performance. Via the GEPIA tool, we acquired the mRNA expression profile of feature genes in SKCM and normal skin tissues from TCGA and GTEx database, and the differential expression was preliminarily validated. The results indicated the differential expression status of AFF1, AHNAK, CAT, CHIN3, COTL1, CXCL9, IFI16, IRF6, LAMB4, LSAMP, PCOLCE2, PLAT, SOX4, and ZBTB16 were confirmed (Fig. 4D).

### Survival analysis and performance evaluation

**Fig. 5:**
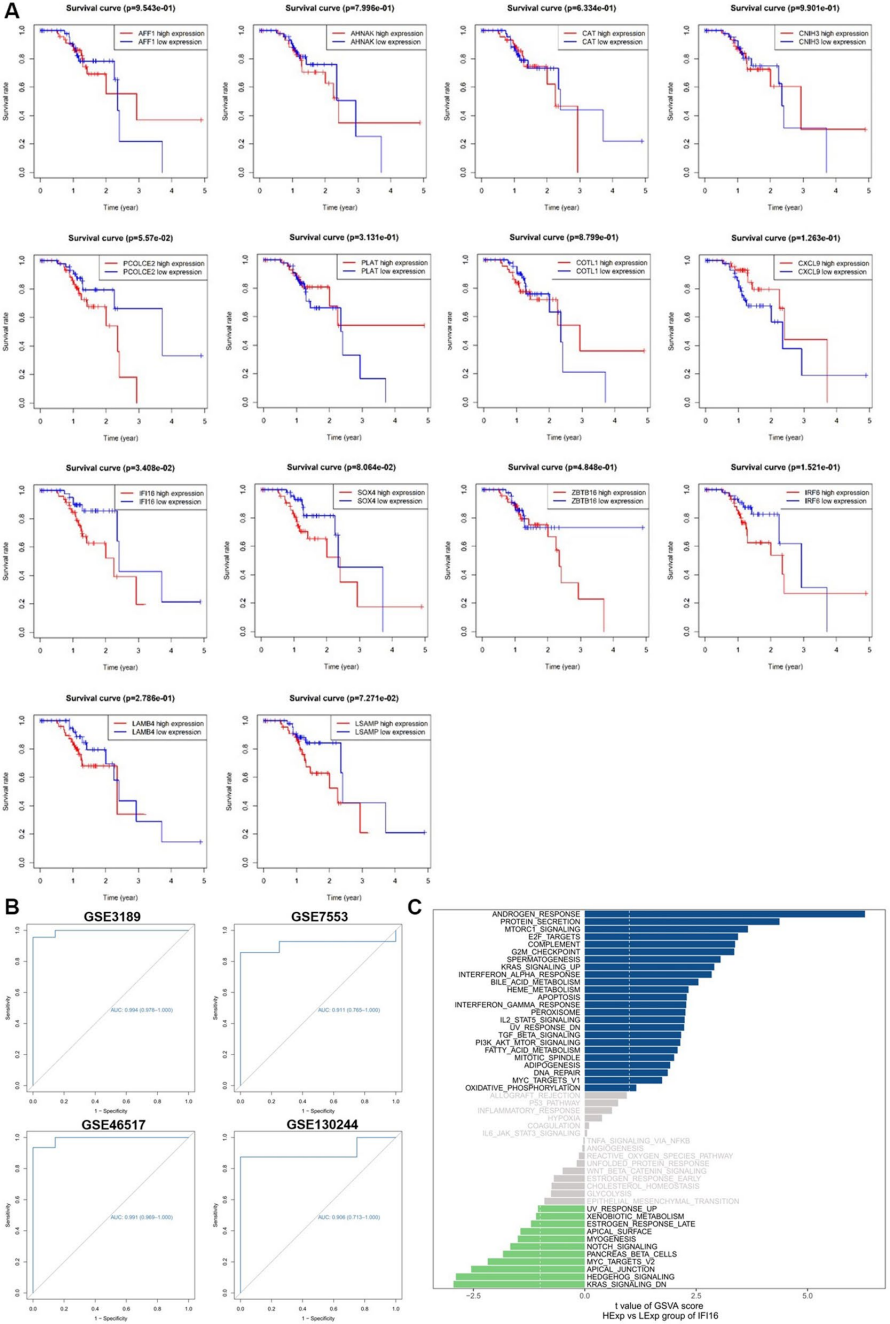
Kaplan–Meier survival analysis of feature genes, the receiver operating characteristic (ROC) curves of the classification effectiveness of the prognostic biomarker IFI16, and the gene set variation analysis of IFI16. **A** Kaplan–Meier survival analysis of AFF1, AHNAK, CAT, CHIN3, COTL1, CXCL9, IFI16, IRF6, LAMB4, LSAMP, PCOLCE2, PLAT, SOX4 and ZBTB16. **B** ROC curves of the classification effectiveness of IFI16 in GSE130244, GSE3189, GSE7553 and GSE46517 datasets. **C** The gene set variation analysis of IFI16; the blue represents the functional annotations of gene sets with up-regulated IFI16, the grey represents the functional annotations of gene sets with no significant difference IFI16, and the green represents the functional annotations of gene sets with down-regulated IFI16

Survival analysis recognized that only IFI16 displayed good prognostic significance in patients with primary melanoma among the 14 validated feature genes. In addition, we also detected that highly expressed IFI16 indicating an unfavorable overall survival time (Fig. 5A). In order to further test the classification efficacy of IFI16, we validated it with the GSE3189, GSE7553, GSE46517 and GSE130244 dataset, respectively. As consequences, AUC = 0.994 in GSE3189, AUC = 0.911 in GSE7553, AUC = 0.991 in GSE46517 and AUC = 0.906 in GSE130244, indicating that the feature selection model had good performance and the results were reliable (Fig. 5B).

### Gene set variation analysis

GSVA was performed to evaluate expression levels of specific pathways and biological process between the gene sets with high and low expression of IFI16. And the results showed that there were 25 entries significantly up-regulated while 12 entries down-regulated via comparison. (Fig. 5C). To further investigate the potential functions of IFI16 in SKCM, the entries of IFI16 high expression group and former GO and KEGG enrichment results were overlapped, and two entries of biological process as “apoptosis (GO:0008630, GO:0097193)”, “ultraviolet response (GO:0009314, GO:0071478)” were both identified in GO analysis and GSVA.

### Correlation analysis between IFI16 and infiltrating immune cells

**Fig. 6:**
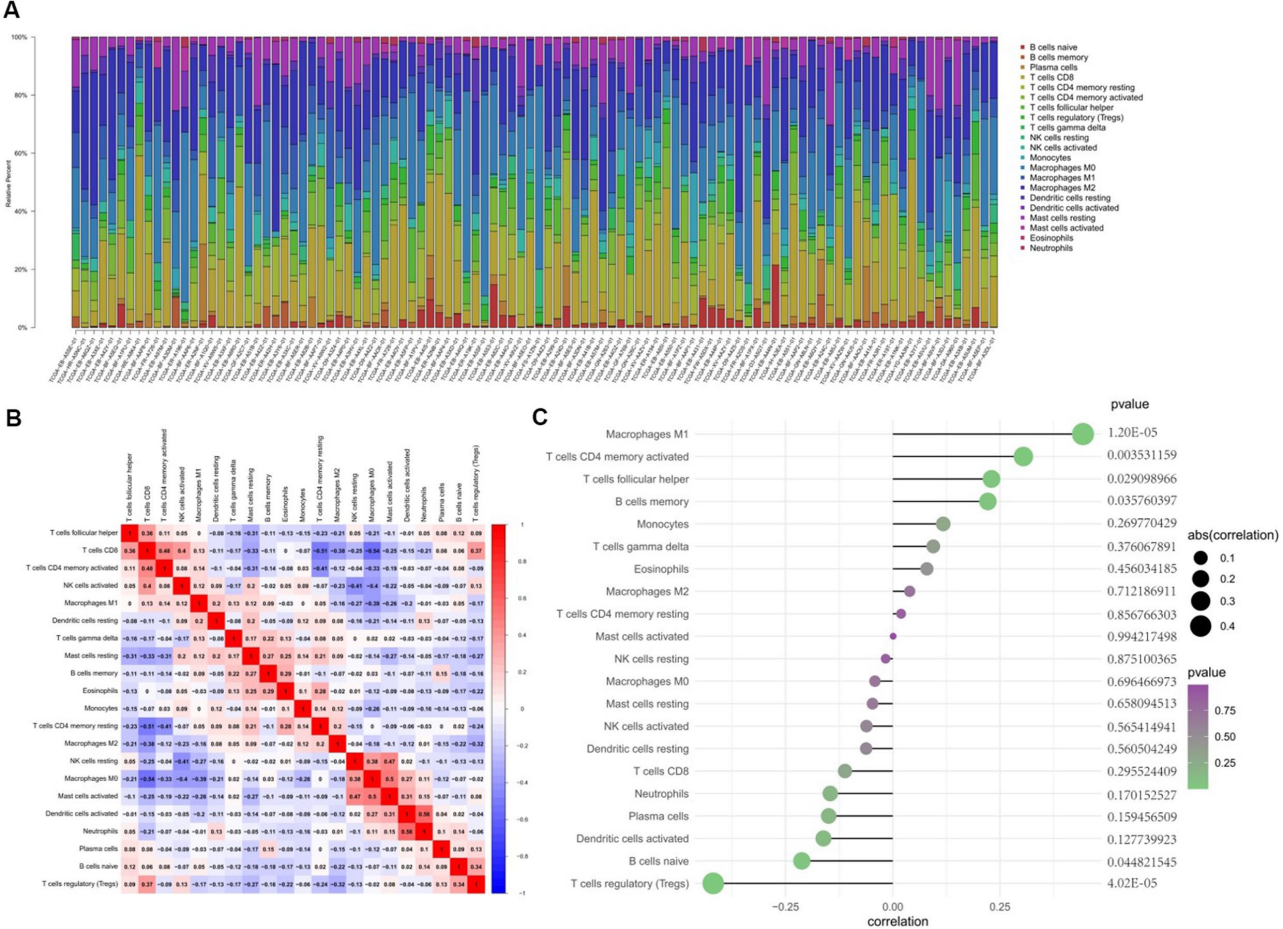
Evaluation of immune cell infiltration and correlation between IFI16 and immune cells. **A** Bar plot of ratio of different immune cells in SKCM samples from TCGA database. **B** Correlation heatmap of immune cells. Red represents positive correlation, blue represents negative correlation, and darker color represents stronger correlation. **C** Correlation between IFI16 and infiltrating immune cells

CIBERSORT algorithm was utilized for exploring the tumor immune microenvironment in primary melanoma. Excluding the non-infiltrating naive CD4^+^ T cells, the proportion of the remaining 21 types of immune cells in the tumor immune microenvironment is shown in Fig. 6A, and Spearman correlation analysis was employed to screen the correlation among the different immune cells (Fig. 6B). Additionally, the correlation between IFI16 and immune cell infiltration was analyzed, and the outcome showed that M1 Macrophages, CD4^+^ activated memory T cells, follicular helper T cells and memory B cells are positively correlated to the expression of IFI16. Meanwhile, regulatory T cells (Tregs) and naive B cells are negatively correlated to the expression of IFI16 (Fig. 6C).

### FISH and IHC validation of IFI16

**Fig. 7:**
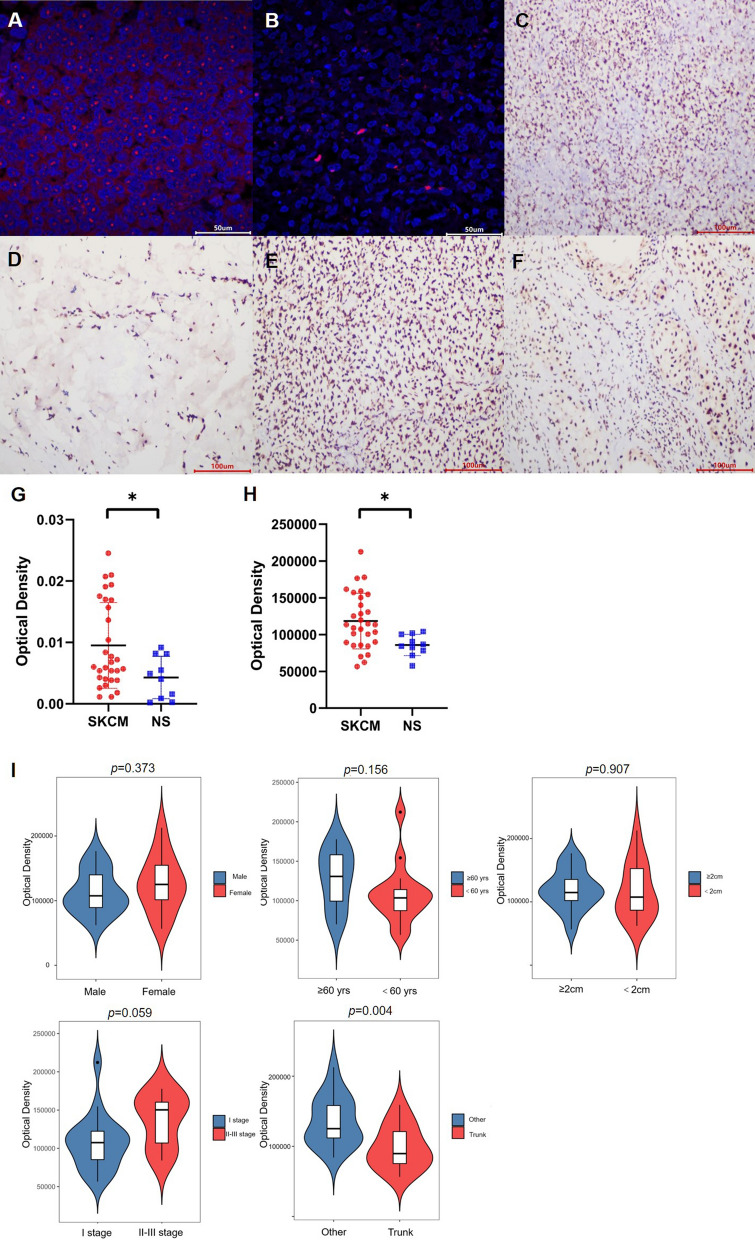
Fluorescence in situ hybridization (FISH) and immunohistochemical (IHC) staining of prognostic biomarker IFI16 in SKCM and normal skin tissue. **A** SKCM (×200) by FISH; **B** normal skin (×200) by FISH; **C** SKCM (×200) by IHC; **D** normal skin (×200) by FISH; **E** SKCM of location outside the trunk (×200) by IHC; **F** SKCM of location inside the trunk (×200) by IHC; **G** comparison of optical density value of FISH; **H** comparison of optical density value of IHC; **I** comparison of optical density value of IHC of SKCM sample with different clinical features

To verify the former bioinformatic analysis results, we tested the mRNA expression of IFI16 by FISH. As shown in Fig. 7G and Table 3, the OD value of SKCM group is significantly higher (*p* < 0.05) than that of normal skin group. Therefore, we can assume that IFI16 was differentially expressed and up-regulated in SKCM, consistent with the former bioinformatic analysis.

**Table 3:** Comparison of mRNA expression levels of IFI16 in SKCM and normal skin tissues

Group	n	OD value	*p*-value
			0.0118
SKCM	30	118,403.3 ± 37,833.8	
Normal skin	10	53,757.5 ± 7709.8	

IHC was applied for protein expression testing, and the OD value of SKCM group is also significantly higher (*p* < 0.05) than that of normal skin group, suggesting higher IFI16 protein expression in SKCM tissue (Fig. 7H, Table 4) which is consistent with our previous consequences. Furthermore, the association between the protein expression level of IFI16 and clinical features was explored. IFI16 protein expression level is correlated with tumor location (*p* < 0.05). There is lower IFI16 protein expression level (*p* < 0.05) in SKCM sample from the trunk than other locations like limbs or head (Fig. 7I, Table 5).

**Table 4:** Comparison of protein expression levels of IFI16 in SKCM and normal skin tissues

Group	n	OD value	*p*-value
			0.0299
SKCM	30	0.0095 ± 0.0070	
Normal skin	10	0.0043 ± 0.0035

**Table 5:** Association between IFI16 protein expression level and clinical features of SKCM patients

	n	OD value	*p*-value
Gender			0.373
Male	17	112,913.9 ± 33,026.3	
Female	13	125,581.8 ± 43,662.1	
Age (years)			0.156
< 60	15	108,528.9 ± 37,743.4	
≥ 60	15	128,277.7 ± 36,490.1	
Diameter (cm)			0.907
< 2	15	117,571.7 ± 44,121.3	
≥ 2	15	119,234.9 ± 31,888.2	
Stage			0.059
Stage I	19	108,549.9 ± 36,777.9	
Stage II–III	11	135,422.8 ± 34,831.7	
Location			0.004
Trunk	14	98,260.1 ± 30,641.7	
Other	16	136,028.6 ± 35,264.4	

## Discussion

SKCM is a highly malignant tumor derived from pigmented cells, causing a large number of disables and deaths every year. The etiology of SKCM is complex, and it is not completely clear. At present, researchers believe that DNA damage of skin pigment cells is the primary cause, and high-risk factors such as ultraviolet damage, radiation, previous malignancy, trauma stimulation, family history are the inducements of canceration [24, 25]. The current diagnostic methods of cutaneous melanoma are reliable, including dermoscopy, ultrasound, and biopsy [2, 26]. The recent advancement of anti-BRAF and anti-MEK targeted therapies has been a significant improvement in treating BRAF*-*mutated patients. However, NRAS-mutated, C-KIT-mutated, and triple-negative patients are still expecting more effective target therapies, and they are currently treated only with immunotherapy or chemotherapy [27]. Meanwhile, because of rapid development and invasiveness, the average survival time of patients with advanced SKCM remains very low. Therefore, discoveries of new genetic signatures of the disease are essential for developing novel therapeutic targets and improving prognosis. In recent years, machine learning has been introduced into bioinformatics, and the immune microenvironment has become a novel direction of cancer research [28, 29]. For example, Yang et al [30] used LASSO regression to identify the valuable immune cells in digestive system tumor, providing novel diagnostic and prognostic biomarkers. Zheng et al. [31] identified immune-related prognostic genes by constructing machine learning model, which might help the progress of immunotherapy of lung carcinoma.

In the present study, we collected four cohorts from the GEO database and carried out a comprehensive analysis. A total of 823 DEGs were screened, including 283 up-regulated and 540 down-regulated genes. The enrichment analysis showed that functions of DEGs mainly clustered in skin development and differentiation, indicating that the process of SKCM accompanying skin cell proliferation, which is as acknowledged. The results of pathway enrichment analysis mainly involved tumor pathways and melanin metabolism pathways, such as tyrosine metabolism, phenylalanine metabolism, PPAR signaling pathway, and tumor associated pathways. In melanocytes, phenylalanine can be transformed into tyrosine via hydroxylation, and then melanin is finally generated with the help of tyrosinase [32]. In the previous study, PPAR ligands and some factors affecting PPAR signaling pathway have been proved to be related to cell proliferation, differentiation, tumor promotion, apoptosis and inflammation [33]. Besides, DEGs enriched in prostate cancer and bladder cancer associated pathways, suggesting that that the pathogenesis of SKCM might be similar to the above diseases.

Based on two feature selection algorithms, 16 feature genes were initially screened. GEPIA contains the gene expression profiling of TCGA and GTEx database, and it was used for preliminary validation of the differential expression of feature genes. At the same time, Kaplan–Meier survival analysis was applied to identify the prognostic value of feature genes, and one potential prognostic biomarker was identified. Interferon gamma inducible protein 16 (IFI16), a member of the HIN-200 family of cytokines, has been reported that it can be induced by interferon as well as environmental factors such as ultraviolet radiation, virus and hypoxia [34]. IFI16 can participate in cell growth and cycle regulation, maintaining physiological function of cells by blocking cell cycle progression, promoting cell aging and apoptosis [35, 36]. Previous studies have found that IFI16 plays a significant role in immune response, and its expression is closely related to the occurrence and development of various tumors [37]. For example, bioinformatic studies have confirmed that IFI16 is a valuable prognostic marker and a promising therapeutic target in renal carcinoma [38]. Also, recent research confirmed that in the pathogenesis of skin squamous cell carcinoma, IFI16, which originally inhibited cell growth, suppressed its expression by methylation and acetylation and reversely promoted tumor growth [39]. In our study, IFI16 gene is up-regulated in SKCM, which is obviously contrary to above mechanism. In addition, research has discovered that IFI16 in tumor cells can bind to p53 protein or downstream proteins such as E2F1 and BRAC1, thus activating p53 related signaling pathway and inducing apoptosis [40]. However, IFI16 and p53 do not always play synergistically. More and more studies have proved that IFI16 can activate NF-κB pathway in p53 inactivated tumor cells, promoting cell growth and playing the role of proto-oncogene [41]. On the contrary, in the cells with regular p53 activity, IFI16 inhibits NF-κB expression, activates the apoptotic protein kinase, and promotes cell apoptosis, playing the role of tumor suppressor gene [42]. As GSVA results indicated, IFI16 participates in the biological process of ultraviolet radiation response and apoptosis in SKCM. As acknowledged, apoptosis plays a critical role in both carcinogenesis and cancer treatment, and problems can arise in any one step along the way of apoptosis [43]. Moreover, we have recognized that ultraviolet radiation is not only an inducing factor of IFI16 but also a high-risk pathogenic factor of SKCM. Therefore, it is reasonable to speculate that ultraviolet radiation may affect the expression of IFI16 and then influence SKCM through the above similar apoptosis mechanism. However, so far, there is no literature on the specific biological role of IFI16 in SKCM.

The immune cell infiltration in SKCM samples was assessed using CIBERSORT. The abnormal distribution and proportion of immune cells in the microenvironment might lead to immune escape, drug resistance, and tumor metastasis. Macrophages can achieve phagocytosis of dead melanoma cells and present cancer antigens that activate secondary adaptive immune responses [44]. T effector cells recognize antigens presented by immune cells via MHC class I molecules and induce cytotoxic effect in melanoma cells [44]. T helper cells bind to the antigen-presenting cells and secret cytokines, eventually leading to tumor cell death [45]. On the contrary, Tregs enhance the secretion of cytokines and chemokines with immunosuppressive activity [46]. The present study found there was positive correlation between IFI16 and M1 Macrophages, CD4^+^ activated memory T cells, follicular helper T cells, and negative correlation between IFI16 and Tregs, suggesting the highly expressed IFI16 might have potential effect on immune response of SKCM.

The results of FISH and IHC experiments suggested expression levels of IFI16 gene and protein were higher in SKCM tissue than normal skin tissue, consistent with the previous prediction. The IHC staining also demonstrated that IFI16 expression level was significantly correlated with the location of tumor (*p* < 0.05). The expression level was significantly higher in tumor occurs on the face, neck, and limbs, which were directly exposed to sunlight, than that of trunk part, which was less vulnerable to sunlight and ultraviolet radiation. Previous studies have shown that ultraviolet radiation was not only a high-risk environmental factor of SKCM but also an important factor affecting the expression of IFI16 [34]. Therefore, we could reasonably speculate that ultraviolet radiation may influence the occurrence of SKCM by affecting the expression level of IFI16 and changing the biological process of cell apoptosis. In our research, the Kaplan–Meier plotter showed IFI16 was significantly associated with patient prognosis. Similarly, previous studies reported that the location of the tumor is significantly related to the survival time of patients with SKCM, consistent with our results [47].

This study employed machine learning methods and Kaplan–Meier survival analysis to screen IFI16 as a signature related to overall survival of SKCM. In addition, GSVA was used for functional annotation and CIBERSORT for analysis of immune cell infiltration in SKCM tissues. At last, FISH and IHC both validated the bioinformatic analysis results. We confirmed that IFI16 is highly expressed in SKCM and associated with poor prognosis of patients, providing a novel potential therapeutic target for SKCM. However, certain limitations should be acknowledged. First of all, as an analysis based on datamining of previously published resource, although some previous research results are consistent with our analysis, more centers and larger samples of clinical data are needed for enhancing reliability. Secondly, more cellular and animal experiments are in need for revealing the function and mechanism of IFI16 in SKCM.

## Conclusions

In summary, the present research identified IFI16 as a signature that can predict the overall survival and affect the tumor immune microenvironment of SKCM. In addition, enrichment analysis showed that IFI16 might play a critical role in the process of SKCM via ultraviolet radiation response and apoptosis. Our current findings provided novel insights into the molecular mechanism of SKCM and potential therapeutic targets. However, further experiments and more extensive clinical samples are required to deeply validate and explore the role of IFI16 in SKCM.

## Supplementary Information

**Additional file 1: Table S1.** SKCM patient information from the TCGA database. **Table S2. **Clinical features of SKCM samples. **Table S3. **Gene Ontology functional enrichment analysis of differentially expressed genes associated with SKCM.

## Data Availability

The datasets used and/or analyzed during the current study are available from the corresponding author upon reasonable request.

## Abbreviations

SKCM: Skin cutaneous melanoma
DEGs: Differentially expressed genes
GSVA: Gene Set Variation Analysis
FISH: Fluorescence in situ hybridization
IHC: Immunohistochemistry
AUC: Area under the receiver operating characteristic curve
ROC: Receiver operating characteristic curve
GEO: Gene Expression Omnibus
GO: Gene ontology
KEGG: Kyoto Encyclopedia of Genes and Genomes
LASSO: Least absolute shrinkage and selection operator
BP: Biological process
MF: Molecular function
CC: Cell component
OD: Optical density
