# Supplementary material for: Comprehensive analysis identifies IFI16 as a novel signature associated with overall survival and immune infiltration of skin cutaneous melanoma
Source: Cancer Cell Int. 2021 Dec 20;21:694. doi: 10.1186/s12935-021-02409-6 (PMC8690488; doi:10.1186/s12935-021-02409-6)
Supplement: Supplementary file 1 — Additional file 1: Table S1. SKCM patient information from the TCGA database. Table S2. Clinical features of SKCM samples. Table S3. Gene Ontology functional enrichment analysis of differentially expressed genes associated with SKCM. [file 12935_2021_2409_MOESM1_ESM.docx]

**Table S1.** SKCM patient information from the TCGA database

| id | futime | fustat | age | gender | stage | T | M | N |
| --- | --- | --- | --- | --- | --- | --- | --- | --- |
| TCGA-EB-A5SE | 401 | 1 | 73 | M | IIB | T3b | M0 | NX |
| TCGA-HR-A5NC | 0 | 0 | 90 | F | IIC | T4 | M0 | NX |
| TCGA-EB-A6QZ | 352 | 1 | 76 | F | IIA | T3a | M0 | N0 |
| TCGA-EB-A3XE | 180 | 0 | 77 | F | IIA | T3a | M0 | N0 |
| TCGA-EB-A42Y | 440 | 0 | 73 | F | IIC | T4b | M0 | N0 |
| TCGA-BF-A5EQ | 323 | 0 | 63 | M | IIC | T4b | M0 | N0 |
| TCGA-BF-A1PU | 387 | 0 | 46 | F | IIC | T4b | M0 | N0 |
| TCGA-WE-A8K4 | 509 | 0 | 85 | M | IIB | T4a | M0 | NX |
| TCGA-BF-AAP8 | 447 | 0 | 58 | M | IIC | T4b | M0 | N0 |
| TCGA-FR-A726 | 0 | 0 | 90 | M | IIC | T4b | M0 | N0 |
| TCGA-EB-A97M | 414 | 0 | 66 | M | IIC | T4b | M0 | N0 |
| TCGA-BF-A3DM | 601 | 0 | 63 | M | IIA | T2b | M0 | N0 |
| TCGA-ER-A196 | 1785 | 0 | 64 | F | IIC | T4b | M0 | N0 |
| TCGA-BF-AAP6 | 325 | 0 | 55 | M | III | T4b | M0 | N2 |
| TCGA-ER-A2NF | 877 | 1 | 53 | M | IIIB | T3b | M0 | N3 |
| TCGA-BF-A1Q0 | 831 | 0 | 80 | M | IIC | T4b | M0 | N0 |
| TCGA-XV-A9W5 | 392 | 0 | 51 | M | I/II | T2 | M0 | N0 |
| TCGA-EB-A3XF | 278 | 0 | 57 | M | IIC | T4b | M0 | N0 |
| TCGA-EB-A6R0 | 467 | 0 | 58 | F | IIC | T4b | M0 | N0 |
| TCGA-GF-A2C7 | 21 | 0 | 48 | M | IIC | T4b | M0 | N0 |
| TCGA-EB-A51B | 537 | 0 | 53 | M | IIC | T4b | M0 | NX |
| TCGA-EB-A42Z | 441 | 0 | 49 | M | IIIC | T4b | M0 | N1b |
| TCGA-ER-A42H | 426 | 1 | 76 | M | - | - | - | - |
| TCGA-EB-A3Y6 | 126 | 0 | 56 | F | IIC | T4b | M0 | N0 |
| TCGA-EB-A3XC | 650 | 0 | 74 | M | IIC | T4b | M0 | N0 |
| TCGA-BF-A5ER | 327 | 0 | 63 | M | IIC | T4b | M0 | N0 |
| TCGA-EB-A82B | 390 | 0 | 58 | F | III | T4b | M0 | N2 |
| TCGA-BF-AAP7 | 318 | 0 | 76 | F | IIC | T4b | M0 | N0 |
| TCGA-XV-A9W2 | 417 | 0 | 81 | M | I | T1 | M0 | N0 |
| TCGA-D9-A3Z4 | 519 | 1 | 54 | M | IIIC | T4b | M0 | N3 |
| TCGA-EB-A4P0 | 326 | 1 | 82 | M | IIC | T4b | M0 | N0 |
| TCGA-EB-A3HV | 39 | 0 | 37 | M | IIC | T4b | M0 | N0 |
| TCGA-EB-A4XL | 777 | 0 | 56 | F | IIC | T4b | M0 | NX |
| TCGA-D9-A4Z2 | 190 | 1 | 50 | M | IIIC | T4b | M0 | N3 |
| TCGA-BF-AAOX | 444 | 0 | 83 | M | IIC | T4b | M0 | N0 |
| TCGA-FR-A728 | 583 | 0 | 54 | F | IIIB | T4b | M0 | N2a |
| TCGA-EB-A4OY | 593 | 0 | 65 | F | IIIB | T4b | M0 | N1a |
| TCGA-EB-A5FP | 454 | 1 | 65 | F | IV | T4b | M1b | NX |
| TCGA-BF-A1PV | 14 | 0 | 74 | F | IIC | T4b | M0 | N0 |
| TCGA-EB-A4IS | 405 | 0 | 77 | M | IIB | T3b | M0 | NX |
| TCGA-ER-A2NB | 857 | 1 | 57 | M | IIIB | T4b | M0 | N2 |
| TCGA-BF-AAP4 | 335 | 0 | 61 | M | IIC | T4b | M0 | N0 |
| TCGA-EB-A3XD | 430 | 0 | 53 | F | IIC | T4b | M0 | NX |
| TCGA-EB-A4IQ | 414 | 0 | 42 | F | IIIB | T4b | M0 | N1 |
| TCGA-ER-A19K | 469 | 1 | 79 | F | IIC | T4b | M0 | N0 |
| TCGA-EB-A5SF | 369 | 1 | 78 | F | IIC | T4b | M0 | NX |
| TCGA-EB-A553 | 226 | 0 | 62 | M | IIC | T4b | M0 | N0 |
| TCGA-EB-A82C | 17 | 0 | 70 | F | IIC | T4b | M0 | N0 |
| TCGA-EB-A44O | 81 | 0 | 69 | M | IIB | T4a | M0 | N0 |
| TCGA-XV-A9VZ | 0 | 0 | 90 | F | II | T4 | M0 | N0 |
| TCGA-BF-A5EO | 703 | 0 | 65 | M | IIC | T4b | M0 | N0 |
| TCGA-FS-A1ZN | 730 | 1 | 43 | M | IIIA | T4b | M0 | N1a |
| TCGA-D9-A4Z3 | 505 | 0 | 73 | F | IIIC | T4b | M0 | N1b |
| TCGA-EB-A299 | 378 | 0 | 63 | M | IIA | T2b | M0 | N0 |
| TCGA-EB-A24D | 645 | 0 | 72 | M | IIIB | T4a | M0 | N2b |
| TCGA-BF-A5ES | 490 | 0 | 76 | F | IIC | T4b | M0 | N0 |
| TCGA-BF-A3DN | 717 | 0 | 81 | F | IIIC | T3b | M0 | N3 |
| TCGA-EB-A41B | 291 | 0 | 76 | F | IIC | T4b | M0 | N0 |
| TCGA-EB-A57M | 399 | 0 | 56 | M | IIIB | T4b | M0 | N1 |
| TCGA-GN-A263 | 467 | 1 | 24 | M | IV | T4b | M1c | N3 |
| TCGA-EB-A4OZ | 620 | 0 | 41 | F | IIIC | T4a | M0 | N3 |
| TCGA-GF-A769 | 1070 | 1 | 39 | M | IIC | T4b | M0 | NX |
| TCGA-GN-A26C | 821 | 1 | 77 | M | IIIC | T4b | M0 | N2b |
| TCGA-XV-AAZV | 412 | 0 | 56 | F | II | T4 | M0 | N0 |
| TCGA-ER-A194 | 1354 | 1 | 77 | M | - | - | M0 | N0 |
| TCGA-EB-A85I | 362 | 0 | 66 | M | IIC | T4b | M0 | N0 |
| TCGA-EB-A550 | 264 | 1 | 75 | F | IIC | T4b | M0 | NX |
| TCGA-BF-A1PZ | 853 | 0 | 71 | F | IIB | T4a | M0 | N0 |
| TCGA-BF-AAP1 | 409 | 0 | 86 | M | IIC | T4b | M0 | N0 |
| TCGA-EB-A431 | 568 | 0 | 34 | M | IIC | T4b | M0 | N0 |
| TCGA-FW-A5DX | 640 | 0 | 71 | M | IIIC | T4a | - | N3 |
| TCGA-EB-A44P | 376 | 0 | 58 | F | IIC | T4b | M0 | N0 |
| TCGA-XV-AAZY | 405 | 0 | 76 | F | IIIC | T4 | M0 | N3 |
| TCGA-D9-A4Z5 | 218 | 0 | 68 | M | IIB | T4a | M0 | N0 |
| TCGA-FR-A2OS | 368 | 1 | 49 | F | IIC | T4b | M0 | N0 |
| TCGA-BF-A1PX | 282 | 1 | 56 | M | IIIB | T4b | M0 | N2a |
| TCGA-D3-A5GT | 487 | 0 | 43 | M | IIIC | T2b | M0 | N3 |
| TCGA-EB-A44N | 205 | 1 | 59 | M | IIC | T4b | M0 | N0 |
| TCGA-IH-A3EA | 524 | 0 | 61 | M | IIC | T4b | M0 | N0 |
| TCGA-EB-A551 | 590 | 0 | 78 | F | IIIC | T4b | M0 | N2b |
| TCGA-BF-AAP2 | 405 | 0 | 62 | M | IIB | T3b | M0 | N0 |
| TCGA-GN-A8LN | 603 | 0 | 68 | M | IIC | T4b | M0 | NX |
| TCGA-EB-A6QY | 382 | 0 | 71 | M | IIC | T4b | M0 | N0 |
| TCGA-EB-A24C | 632 | 0 | 56 | M | IIC | T4b | M0 | N0 |
| TCGA-EB-A85J | 360 | 0 | 66 | F | IIB | T4a | M0 | N0 |
| TCGA-XV-AAZW | 393 | 1 | 62 | F | II | T4 | M0 | N0 |
| TCGA-GN-A4U5 | 1156 | 0 | 61 | F | IB | T2a | M0 | NX |
| TCGA-BF-AAOU | 476 | 0 | 73 | F | IIC | T4b | M0 | N0 |
| TCGA-EB-A41A | 0 | 0 | 90 | M | IIC | T4b | M0 | N0 |
| TCGA-FR-A3R1 | 685 | 0 | 69 | M | IIC | T4b | M0 | N0 |
| TCGA-ER-A19T | 270 | 1 | 51 | M | IV | T4a | M1a | N3 |
| TCGA-EB-A1NK | 613 | 0 | 48 | M | IIC | T4b | M0 | N0 |
| TCGA-YG-AA3N | 306 | 0 | 67 | M | IIC | T4b | M0 | N0 |
| TCGA-EB-A3Y7 | 326 | 1 | 86 | F | IIIB | T3a | M0 | N2c |
| TCGA-EB-A5VU | 321 | 1 | 56 | M | IIIB | T4b | M0 | N1 |
| TCGA-BF-A9VF | 440 | 0 | 77 | M | IIC | T4b | M0 | N0 |
| TCGA-BF-A3DJ | 464 | 0 | 36 | F | IIIB | T4b | M0 | N1 |
| TCGA-DA-A960 | 804 | 0 | 73 | M | IIB | T3b | M0 | N0 |
| TCGA-EB-A3XB | 408 | 0 | 63 | M | II | T4 | M0 | NX |
| TCGA-EB-A5UM | 414 | 0 | 48 | F | IIC | T4b | M0 | N0 |
| TCGA-BF-A5EP | 335 | 0 | 75 | F | IIIC | T4b | M0 | N3 |
| TCGA-BF-A3DL | 769 | 0 | 84 | F | IIIB | T3b | M0 | N2 |

**Table S2.** Clinical features of SKCM samples

|  | Gender | Age  (year) | Diameter（cm） | Location | Pathological Stage | TMN Stage |
| --- | --- | --- | --- | --- | --- | --- |
| 1 | Male | 37 | 1.5 | Trunk | IIB | T3bN0M0 |
| 2 | Female | 67 | 0.8 | Trunk | IA | T1bN0M0 |
| 3 | Male | 74 | 1.5 | Other | IIIB | T3aN1aM0 |
| 4 | Female | 71 | 5 | Other | IIA | T3aN0M0 |
| 5 | Female | 53 | 1.5 | Other | IA | T1aN0M0 |
| 6 | Female | 58 | 5 | Trunk | IA | T1bN0M0 |
| 7 | Male | 68 | 7 | Trunk | IIB | T3bN0M0 |
| 8 | Female | 66 | 6 | Other | IIA | T2bN0M0 |
| 9 | Male | 73 | 1.5 | Trunk | IB | T2aN0M0 |
| 10 | Male | 40 | 1 | Other | IA | T1bN0M0 |
| 11 | Male | 34 | 1.8 | Trunk | IA | T1bN0M0 |
| 12 | Male | 70 | 1.5 | Other | IIB | T3bN0M0 |
| 13 | Female | 39 | 5 | Trunk | IA | T1bN0M0 |
| 14 | Female | 62 | 1.2 | Trunk | IA | T1aN0M0 |
| 15 | Male | 53 | 4 | Other | IIB | T3bN0M0 |
| 16 | Male | 65 | 1.8 | Trunk | IA | T1aN0M0 |
| 17 | Male | 37 | 9.5 | Trunk | IA | T1bN0M0 |
| 18 | Female | 62 | 1.5 | Other | IIA | T2bN0M0 |
| 19 | Male | 23 | 1.5 | Other | IIA | T3aN0M0 |
| 20 | Female | 61 | 2.5 | Other | IA | T1bN0M0 |
| 21 | Male | 67 | 2.5 | Trunk | IA | T1bN0M0 |
| 22 | Female | 66 | 1.5 | Trunk | IA | T1bN0M0 |
| 23 | Male | 78 | 2.5 | Other | IIB | T3bN0M0 |
| 24 | Female | 49 | 0.7 | Other | IA | T1aN0M0 |
| 25 | Male | 41 | 2.2 | Trunk | IA | T1bN0M0 |
| 26 | Female | 55 | 1 | Other | IA | T1bN0M0 |
| 27 | Female | 73 | 3.5 | Trunk | IB | T2aN0M0 |
| 28 | Male | 20 | 3 | Other | IA | T1bN0M0 |
| 29 | Male | 20 | 3 | Other | IIA | T3aN0M0 |
| 30 | Male | 20 | 3 | Other | IB | T2aN0M0 |

**Table S3.** Gene Ontology functional enrichment analysis of differentially expressed genes associated with SKCM.

| ONTOLOGY | ID | Description | *p*-value | Count |
| --- | --- | --- | --- | --- |
| BP | GO:0008544 | epidermis development | 4.08E-25 | 75 |
| BP | GO:0043588 | skin development | 2.35E-24 | 70 |
| BP | GO:0070268 | cornification | 7.36E-18 | 31 |
| BP | GO:0030216 | keratinocyte differentiation | 2.48E-17 | 50 |
| BP | GO:0009913 | epidermal cell differentiation | 5.57E-17 | 54 |
| BP | GO:0001655 | urogenital system development | 6.88E-16 | 50 |
| BP | GO:0030198 | extracellular matrix organization | 5.69E-14 | 50 |
| BP | GO:0043062 | extracellular structure organization | 6.33E-14 | 50 |
| BP | GO:0048871 | multicellular organismal homeostasis | 1.63E-13 | 58 |
| BP | GO:0072001 | renal system development | 2.40E-13 | 43 |
| BP | GO:0061436 | establishment of skin barrier | 5.78E-13 | 13 |
| BP | GO:0031424 | keratinization | 1.44E-12 | 36 |
| BP | GO:0033561 | regulation of water loss via skin | 4.69E-12 | 13 |
| BP | GO:0048732 | gland development | 2.99E-11 | 50 |
| BP | GO:0001822 | kidney development | 5.29E-11 | 38 |
| BP | GO:1901654 | response to ketone | 5.30E-11 | 31 |
| BP | GO:0008217 | regulation of blood pressure | 5.60E-11 | 30 |
| BP | GO:0048608 | reproductive structure development | 7.58E-11 | 49 |
| BP | GO:0030104 | water homeostasis | 7.64E-11 | 19 |
| BP | GO:0061458 | reproductive system development | 9.69E-11 | 49 |
| BP | GO:0050891 | multicellular organismal water homeostasis | 1.39E-10 | 18 |
| BP | GO:0048545 | response to steroid hormone | 1.66E-09 | 43 |
| BP | GO:0002237 | response to molecule of bacterial origin | 2.07E-09 | 40 |
| BP | GO:0032496 | response to lipopolysaccharide | 2.25E-09 | 39 |
| BP | GO:0010951 | negative regulation of endopeptidase activity | 2.45E-09 | 33 |
| BP | GO:0042476 | odontogenesis | 3.25E-09 | 23 |
| BP | GO:0052547 | regulation of peptidase activity | 3.56E-09 | 47 |
| BP | GO:0052548 | regulation of endopeptidase activity | 4.41E-09 | 45 |
| BP | GO:0042445 | hormone metabolic process | 6.16E-09 | 31 |
| BP | GO:0010466 | negative regulation of peptidase activity | 8.04E-09 | 33 |
| BP | GO:0050673 | epithelial cell proliferation | 8.43E-09 | 45 |
| BP | GO:0043616 | keratinocyte proliferation | 8.70E-09 | 13 |
| BP | GO:0031960 | response to corticosteroid | 9.02E-09 | 25 |
| BP | GO:0003014 | renal system process | 1.44E-08 | 21 |
| BP | GO:0071466 | cellular response to xenobiotic stimulus | 1.87E-08 | 26 |
| BP | GO:0001101 | response to acid chemical | 2.14E-08 | 38 |
| BP | GO:0022407 | regulation of cell-cell adhesion | 2.14E-08 | 42 |
| BP | GO:0048565 | digestive tract development | 2.21E-08 | 22 |
| BP | GO:0002064 | epithelial cell development | 2.32E-08 | 28 |
| BP | GO:0051384 | response to glucocorticoid | 2.37E-08 | 23 |
| BP | GO:0055123 | digestive system development | 2.37E-08 | 23 |
| BP | GO:0001823 | mesonephros development | 2.45E-08 | 19 |
| BP | GO:0003073 | regulation of systemic arterial blood pressure | 3.06E-08 | 18 |
| BP | GO:0014074 | response to purine-containing compound | 3.51E-08 | 23 |
| BP | GO:0001503 | ossification | 4.69E-08 | 41 |
| BP | GO:0045861 | negative regulation of proteolysis | 6.68E-08 | 38 |
| BP | GO:0007565 | female pregnancy | 7.17E-08 | 26 |
| BP | GO:0050678 | regulation of epithelial cell proliferation | 9.59E-08 | 39 |
| BP | GO:0046683 | response to organophosphorus | 1.05E-07 | 21 |
| BP | GO:0044706 | multi-multicellular organism process | 1.06E-07 | 28 |
| BP | GO:0009410 | response to xenobiotic stimulus | 1.13E-07 | 33 |
| BP | GO:0040013 | negative regulation of locomotion | 1.18E-07 | 39 |
| BP | GO:0051271 | negative regulation of cellular component movement | 1.45E-07 | 39 |
| BP | GO:0030282 | bone mineralization | 1.57E-07 | 19 |
| BP | GO:1901655 | cellular response to ketone | 1.77E-07 | 17 |
| BP | GO:0030278 | regulation of ossification | 2.21E-07 | 26 |
| BP | GO:0001890 | placenta development | 2.23E-07 | 22 |
| BP | GO:0061448 | connective tissue development | 2.46E-07 | 31 |
| BP | GO:0098742 | cell-cell adhesion via plasma-membrane adhesion molecules | 2.46E-07 | 31 |
| BP | GO:2000146 | negative regulation of cell motility | 3.00E-07 | 36 |
| BP | GO:0003071 | renal system process involved in regulation of systemic arterial blood pressure | 3.25E-07 | 9 |
| BP | GO:0001657 | ureteric bud development | 3.33E-07 | 17 |
| BP | GO:0051591 | response to cAMP | 3.33E-07 | 17 |
| BP | GO:0006631 | fatty acid metabolic process | 3.80E-07 | 38 |
| BP | GO:0043473 | pigmentation | 3.88E-07 | 17 |
| BP | GO:0072163 | mesonephric epithelium development | 3.88E-07 | 17 |
| BP | GO:0072164 | mesonephric tubule development | 3.88E-07 | 17 |
| BP | GO:0042119 | neutrophil activation | 4.77E-07 | 45 |
| BP | GO:0007160 | cell-matrix adhesion | 4.90E-07 | 27 |
| BP | GO:0060541 | respiratory system development | 4.95E-07 | 25 |
| BP | GO:0031667 | response to nutrient levels | 5.04E-07 | 45 |
| BP | GO:0045104 | intermediate filament cytoskeleton organization | 5.39E-07 | 12 |
| BP | GO:0072593 | reactive oxygen species metabolic process | 5.90E-07 | 31 |
| BP | GO:0045103 | intermediate filament-based process | 6.79E-07 | 12 |
| BP | GO:2000379 | positive regulation of reactive oxygen species metabolic process | 7.00E-07 | 17 |
| BP | GO:0010038 | response to metal ion | 7.28E-07 | 36 |
| BP | GO:0031214 | biomineral tissue development | 7.62E-07 | 22 |
| BP | GO:0110148 | biomineralization | 7.62E-07 | 22 |
| BP | GO:0032963 | collagen metabolic process | 8.65E-07 | 18 |
| BP | GO:0048568 | embryonic organ development | 8.87E-07 | 40 |
| BP | GO:0072073 | kidney epithelium development | 9.70E-07 | 20 |
| BP | GO:0097305 | response to alcohol | 9.80E-07 | 27 |
| BP | GO:1903034 | regulation of response to wounding | 1.03E-06 | 23 |
| BP | GO:0051346 | negative regulation of hydrolase activity | 1.04E-06 | 42 |
| BP | GO:0031589 | cell-substrate adhesion | 1.19E-06 | 35 |
| BP | GO:0050886 | endocrine process | 1.27E-06 | 15 |
| BP | GO:0045785 | positive regulation of cell adhesion | 1.34E-06 | 38 |
| BP | GO:0061138 | morphogenesis of a branching epithelium | 1.39E-06 | 23 |
| BP | GO:0048660 | regulation of smooth muscle cell proliferation | 1.42E-06 | 22 |
| BP | GO:0060135 | maternal process involved in female pregnancy | 1.42E-06 | 13 |
| BP | GO:0001763 | morphogenesis of a branching structure | 1.46E-06 | 24 |
| BP | GO:0001977 | renal system process involved in regulation of blood volume | 1.47E-06 | 7 |
| BP | GO:0033002 | muscle cell proliferation | 1.61E-06 | 27 |
| BP | GO:0022612 | gland morphogenesis | 1.64E-06 | 18 |
| BP | GO:0048659 | smooth muscle cell proliferation | 1.73E-06 | 22 |
| BP | GO:0090183 | regulation of kidney development | 1.98E-06 | 12 |
| BP | GO:0019216 | regulation of lipid metabolic process | 2.03E-06 | 38 |
| BP | GO:0030336 | negative regulation of cell migration | 2.42E-06 | 33 |
| BP | GO:0060326 | cell chemotaxis | 2.53E-06 | 31 |
| BP | GO:0007162 | negative regulation of cell adhesion | 2.55E-06 | 30 |
| BP | GO:0045742 | positive regulation of epidermal growth factor receptor signaling pathway | 2.58E-06 | 9 |
| BP | GO:0042303 | molting cycle | 2.68E-06 | 17 |
| BP | GO:0042633 | hair cycle | 2.68E-06 | 17 |
| BP | GO:0030323 | respiratory tube development | 2.81E-06 | 22 |
| BP | GO:0007568 | aging | 2.84E-06 | 32 |
| BP | GO:0032102 | negative regulation of response to external stimulus | 2.90E-06 | 37 |
| BP | GO:0002446 | neutrophil mediated immunity | 2.97E-06 | 43 |
| BP | GO:0050900 | leukocyte migration | 2.97E-06 | 43 |
| BP | GO:0060562 | epithelial tube morphogenesis | 3.04E-06 | 32 |
| BP | GO:0045778 | positive regulation of ossification | 3.14E-06 | 15 |
| BP | GO:0007584 | response to nutrient | 3.22E-06 | 25 |
| BP | GO:0003044 | regulation of systemic arterial blood pressure mediated by a chemical signal | 3.26E-06 | 11 |
| BP | GO:0003334 | keratinocyte development | 3.29E-06 | 6 |
| BP | GO:0043312 | neutrophil degranulation | 3.42E-06 | 42 |
| BP | GO:0002283 | neutrophil activation involved in immune response | 3.99E-06 | 42 |
| BP | GO:0022617 | extracellular matrix disassembly | 4.28E-06 | 14 |
| BP | GO:0018149 | peptide cross-linking | 4.28E-06 | 12 |
| BP | GO:0071229 | cellular response to acid chemical | 4.55E-06 | 24 |
| BP | GO:1901186 | positive regulation of ERBB signaling pathway | 4.58E-06 | 9 |
| BP | GO:0003206 | cardiac chamber morphogenesis | 4.72E-06 | 18 |
| BP | GO:0070661 | leukocyte proliferation | 4.75E-06 | 30 |
| BP | GO:1901184 | regulation of ERBB signaling pathway | 4.78E-06 | 15 |
| BP | GO:0030595 | leukocyte chemotaxis | 4.83E-06 | 25 |
| BP | GO:0061844 | antimicrobial humoral immune response mediated by antimicrobial peptide | 6.67E-06 | 13 |
| BP | GO:0030324 | lung development | 6.84E-06 | 21 |
| BP | GO:0045682 | regulation of epidermis development | 7.67E-06 | 14 |
| BP | GO:0098801 | regulation of renal system process | 7.80E-06 | 9 |
| BP | GO:0006066 | alcohol metabolic process | 8.38E-06 | 34 |
| BP | GO:0043393 | regulation of protein binding | 8.70E-06 | 24 |
| BP | GO:0016999 | antibiotic metabolic process | 8.78E-06 | 17 |
| BP | GO:0019730 | antimicrobial humoral response | 8.78E-06 | 17 |
| BP | GO:0042058 | regulation of epidermal growth factor receptor signaling pathway | 8.83E-06 | 14 |
| BP | GO:0070542 | response to fatty acid | 8.83E-06 | 14 |
| BP | GO:0035107 | appendage morphogenesis | 8.96E-06 | 19 |
| BP | GO:0035108 | limb morphogenesis | 8.96E-06 | 19 |
| BP | GO:0061041 | regulation of wound healing | 8.96E-06 | 19 |
| BP | GO:0022404 | molting cycle process | 1.16E-05 | 14 |
| BP | GO:0022405 | hair cycle process | 1.16E-05 | 14 |
| BP | GO:0060443 | mammary gland morphogenesis | 1.24E-05 | 10 |
| BP | GO:0048736 | appendage development | 1.27E-05 | 21 |
| BP | GO:0060173 | limb development | 1.27E-05 | 21 |
| BP | GO:0010959 | regulation of metal ion transport | 1.29E-05 | 35 |
| BP | GO:0045444 | fat cell differentiation | 1.38E-05 | 24 |
| BP | GO:0022408 | negative regulation of cell-cell adhesion | 1.39E-05 | 21 |
| BP | GO:0051216 | cartilage development | 1.45E-05 | 23 |
| BP | GO:2000377 | regulation of reactive oxygen species metabolic process | 1.49E-05 | 22 |
| BP | GO:0042475 | odontogenesis of dentin-containing tooth | 1.51E-05 | 14 |
| BP | GO:1903035 | negative regulation of response to wounding | 1.51E-05 | 14 |
| BP | GO:0048066 | developmental pigmentation | 1.52E-05 | 10 |
| BP | GO:0071902 | positive regulation of protein serine/threonine kinase activity | 1.71E-05 | 31 |
| BP | GO:0072006 | nephron development | 1.83E-05 | 18 |
| BP | GO:0071549 | cellular response to dexamethasone stimulus | 1.88E-05 | 8 |
| BP | GO:0030879 | mammary gland development | 2.01E-05 | 18 |
| BP | GO:0030501 | positive regulation of bone mineralization | 2.02E-05 | 9 |
| BP | GO:0003205 | cardiac chamber development | 2.13E-05 | 20 |
| BP | GO:0002934 | desmosome organization | 2.31E-05 | 5 |
| BP | GO:0016338 | calcium-independent cell-cell adhesion via plasma membrane cell-adhesion molecules | 2.45E-05 | 7 |
| BP | GO:0045109 | intermediate filament organization | 2.45E-05 | 7 |
| BP | GO:0051098 | regulation of binding | 2.46E-05 | 33 |
| BP | GO:0001990 | regulation of systemic arterial blood pressure by hormone | 2.52E-05 | 9 |
| BP | GO:0071548 | response to dexamethasone | 2.52E-05 | 9 |
| BP | GO:0090184 | positive regulation of kidney development | 2.52E-05 | 9 |
| BP | GO:0045598 | regulation of fat cell differentiation | 2.52E-05 | 17 |
| BP | GO:0050920 | regulation of chemotaxis | 2.65E-05 | 23 |
| BP | GO:0034329 | cell junction assembly | 2.84E-05 | 35 |
| BP | GO:0034113 | heterotypic cell-cell adhesion | 3.00E-05 | 11 |
| BP | GO:0045926 | negative regulation of growth | 3.02E-05 | 25 |
| BP | GO:0032526 | response to retinoic acid | 3.05E-05 | 15 |
| BP | GO:0051047 | positive regulation of secretion | 3.16E-05 | 36 |
| BP | GO:1901617 | organic hydroxy compound biosynthetic process | 3.35E-05 | 26 |
| BP | GO:0001889 | liver development | 3.38E-05 | 17 |
| BP | GO:0001676 | long-chain fatty acid metabolic process | 3.40E-05 | 15 |
| BP | GO:0043627 | response to estrogen | 3.47E-05 | 12 |
| BP | GO:0008202 | steroid metabolic process | 3.64E-05 | 30 |
| BP | GO:0034308 | primary alcohol metabolic process | 3.65E-05 | 13 |
| BP | GO:0051235 | maintenance of location | 3.68E-05 | 29 |
| BP | GO:0043651 | linoleic acid metabolic process | 3.70E-05 | 6 |
| BP | GO:0090185 | negative regulation of kidney development | 3.70E-05 | 6 |
| BP | GO:0033559 | unsaturated fatty acid metabolic process | 3.80E-05 | 15 |
| BP | GO:0003093 | regulation of glomerular filtration | 4.10E-05 | 5 |
| BP | GO:0001942 | hair follicle development | 4.14E-05 | 13 |
| BP | GO:0048705 | skeletal system morphogenesis | 4.32E-05 | 24 |
| BP | GO:0008211 | glucocorticoid metabolic process | 4.48E-05 | 7 |
| BP | GO:0061008 | hepaticobiliary system development | 4.49E-05 | 17 |
| BP | GO:0010043 | response to zinc ion | 4.59E-05 | 10 |
| BP | GO:0071320 | cellular response to cAMP | 4.59E-05 | 10 |
| BP | GO:0006805 | xenobiotic metabolic process | 4.67E-05 | 16 |
| BP | GO:0030326 | embryonic limb morphogenesis | 4.67E-05 | 16 |
| BP | GO:0035113 | embryonic appendage morphogenesis | 4.67E-05 | 16 |
| BP | GO:0002065 | columnar/cuboidal epithelial cell differentiation | 4.70E-05 | 15 |
| BP | GO:0097529 | myeloid leukocyte migration | 4.71E-05 | 22 |
| BP | GO:1901568 | fatty acid derivative metabolic process | 4.99E-05 | 19 |
| BP | GO:0003382 | epithelial cell morphogenesis | 5.05E-05 | 8 |
| BP | GO:0030500 | regulation of bone mineralization | 5.24E-05 | 12 |
| BP | GO:0061045 | negative regulation of wound healing | 5.24E-05 | 12 |
| BP | GO:0098773 | skin epidermis development | 5.30E-05 | 13 |
| BP | GO:0006704 | glucocorticoid biosynthetic process | 5.36E-05 | 6 |
| BP | GO:0006636 | unsaturated fatty acid biosynthetic process | 5.43E-05 | 10 |
| BP | GO:0061005 | cell differentiation involved in kidney development | 5.43E-05 | 10 |
| BP | GO:0060675 | ureteric bud morphogenesis | 5.55E-05 | 11 |
| BP | GO:0042063 | gliogenesis | 5.57E-05 | 27 |
| BP | GO:0003081 | regulation of systemic arterial blood pressure by renin-angiotensin | 5.91E-05 | 7 |
| BP | GO:0032602 | chemokine production | 5.98E-05 | 13 |
| BP | GO:0060021 | roof of mouth development | 5.98E-05 | 13 |
| BP | GO:0001894 | tissue homeostasis | 6.18E-05 | 23 |
| BP | GO:0072171 | mesonephric tubule morphogenesis | 6.42E-05 | 11 |
| BP | GO:0038127 | ERBB signaling pathway | 6.47E-05 | 17 |
| BP | GO:0030856 | regulation of epithelial cell differentiation | 6.49E-05 | 18 |
| BP | GO:0045216 | cell-cell junction organization | 6.61E-05 | 20 |
| BP | GO:0019755 | one-carbon compound transport | 6.79E-05 | 5 |
| BP | GO:0031581 | hemidesmosome assembly | 6.79E-05 | 5 |
| BP | GO:0090130 | tissue migration | 7.53E-05 | 31 |
| BP | GO:0003231 | cardiac ventricle development | 7.54E-05 | 16 |
| BP | GO:0071695 | anatomical structure maturation | 7.54E-05 | 23 |
| BP | GO:0071887 | leukocyte apoptotic process | 7.89E-05 | 14 |
| BP | GO:0060711 | labyrinthine layer development | 8.16E-05 | 9 |
| BP | GO:0010001 | glial cell differentiation | 8.25E-05 | 22 |
| BP | GO:2000117 | negative regulation of cysteine-type endopeptidase activity | 8.50E-05 | 13 |
| BP | GO:0048638 | regulation of developmental growth | 8.67E-05 | 30 |
| BP | GO:1903428 | positive regulation of reactive oxygen species biosynthetic process | 8.77E-05 | 10 |
| BP | GO:0007409 | axonogenesis | 9.10E-05 | 37 |
| BP | GO:0003018 | vascular process in circulatory system | 9.43E-05 | 19 |
| BP | GO:0072080 | nephron tubule development | 9.52E-05 | 13 |
| BP | GO:0007173 | epidermal growth factor receptor signaling pathway | 9.54E-05 | 15 |
| BP | GO:0032941 | secretion by tissue | 9.64E-05 | 8 |
| BP | GO:0070169 | positive regulation of biomineral tissue development | 9.73E-05 | 9 |
| BP | GO:0110151 | positive regulation of biomineralization | 9.73E-05 | 9 |
| BP | GO:0070227 | lymphocyte apoptotic process | 9.76E-05 | 11 |
| BP | GO:0060512 | prostate gland morphogenesis | 9.92E-05 | 7 |
| BP | GO:0017001 | antibiotic catabolic process | 0.000102 | 10 |
| BP | GO:0043434 | response to peptide hormone | 0.000104 | 35 |
| BP | GO:0072330 | monocarboxylic acid biosynthetic process | 0.000104 | 23 |
| BP | GO:1900424 | regulation of defense response to bacterium | 0.000104 | 6 |
| BP | GO:0070167 | regulation of biomineral tissue development | 0.000106 | 13 |
| BP | GO:0110149 | regulation of biomineralization | 0.000106 | 13 |
| BP | GO:0060100 | positive regulation of phagocytosis, engulfment | 0.000107 | 5 |
| BP | GO:1905155 | positive regulation of membrane invagination | 0.000107 | 5 |
| BP | GO:0042692 | muscle cell differentiation | 0.000107 | 32 |
| BP | GO:0060070 | canonical Wnt signaling pathway | 0.000111 | 29 |
| BP | GO:1904951 | positive regulation of establishment of protein localization | 0.000116 | 36 |
| BP | GO:0050921 | positive regulation of chemotaxis | 0.000119 | 16 |
| BP | GO:0001658 | branching involved in ureteric bud morphogenesis | 0.000119 | 10 |
| BP | GO:0061326 | renal tubule development | 0.000119 | 13 |
| BP | GO:0007219 | Notch signaling pathway | 0.000119 | 20 |
| BP | GO:0043405 | regulation of MAP kinase activity | 0.000123 | 29 |
| BP | GO:0051222 | positive regulation of protein transport | 0.000124 | 35 |
| BP | GO:0006633 | fatty acid biosynthetic process | 0.000124 | 18 |
| BP | GO:0048730 | epidermis morphogenesis | 0.000126 | 7 |
| BP | GO:1903409 | reactive oxygen species biosynthetic process | 0.000127 | 15 |
| BP | GO:0048771 | tissue remodeling | 0.000127 | 19 |
| BP | GO:0048754 | branching morphogenesis of an epithelial tube | 0.000128 | 17 |
| BP | GO:0046394 | carboxylic acid biosynthetic process | 0.00013 | 30 |
| BP | GO:0001649 | osteoblast differentiation | 0.000131 | 22 |
| BP | GO:0072009 | nephron epithelium development | 0.000132 | 14 |
| BP | GO:0016053 | organic acid biosynthetic process | 0.000137 | 30 |
| BP | GO:0002062 | chondrocyte differentiation | 0.000139 | 15 |
| BP | GO:0042438 | melanin biosynthetic process | 0.000141 | 6 |
| BP | GO:0060713 | labyrinthine layer morphogenesis | 0.000141 | 6 |
| BP | GO:0045445 | myoblast differentiation | 0.000142 | 12 |
| BP | GO:0061180 | mammary gland epithelium development | 0.000145 | 11 |
| BP | GO:0048015 | phosphatidylinositol-mediated signaling | 0.000148 | 19 |
| BP | GO:0016054 | organic acid catabolic process | 0.000151 | 25 |
| BP | GO:0046395 | carboxylic acid catabolic process | 0.000151 | 25 |
| BP | GO:0071219 | cellular response to molecule of bacterial origin | 0.000154 | 21 |
| BP | GO:0042733 | embryonic digit morphogenesis | 0.000158 | 10 |
| BP | GO:0042759 | long-chain fatty acid biosynthetic process | 0.000159 | 7 |
| BP | GO:0042110 | T cell activation | 0.000164 | 36 |
| BP | GO:0003208 | cardiac ventricle morphogenesis | 0.000164 | 11 |
| BP | GO:0046677 | response to antibiotic | 0.000173 | 28 |
| BP | GO:0003279 | cardiac septum development | 0.000177 | 14 |
| BP | GO:0031032 | actomyosin structure organization | 0.000181 | 20 |
| BP | GO:0048857 | neural nucleus development | 0.000182 | 10 |
| BP | GO:0051100 | negative regulation of binding | 0.000182 | 18 |
| BP | GO:0048017 | inositol lipid-mediated signaling | 0.000183 | 19 |
| BP | GO:0072078 | nephron tubule morphogenesis | 0.000186 | 11 |
| BP | GO:0006582 | melanin metabolic process | 0.000187 | 6 |
| BP | GO:0032402 | melanosome transport | 0.000187 | 6 |
| BP | GO:0010810 | regulation of cell-substrate adhesion | 0.000188 | 21 |
| BP | GO:0048640 | negative regulation of developmental growth | 0.000194 | 14 |
| BP | GO:0001818 | negative regulation of cytokine production | 0.000195 | 26 |
| BP | GO:0001892 | embryonic placenta development | 0.000199 | 12 |
| BP | GO:0022600 | digestive system process | 0.000201 | 13 |
| BP | GO:0001558 | regulation of cell growth | 0.000202 | 33 |
| BP | GO:0002688 | regulation of leukocyte chemotaxis | 0.000213 | 14 |
| BP | GO:0060349 | bone morphogenesis | 0.000213 | 14 |
| BP | GO:0016485 | protein processing | 0.000214 | 21 |
| BP | GO:0060348 | bone development | 0.000214 | 21 |
| BP | GO:0048661 | positive regulation of smooth muscle cell proliferation | 0.000222 | 13 |
| BP | GO:0009791 | post-embryonic development | 0.000222 | 12 |
| BP | GO:0060099 | regulation of phagocytosis, engulfment | 0.000233 | 5 |
| BP | GO:0035296 | regulation of tube diameter | 0.000233 | 16 |
| BP | GO:0097746 | regulation of blood vessel diameter | 0.000233 | 16 |
| BP | GO:0106106 | cold-induced thermogenesis | 0.000233 | 16 |
| BP | GO:0120161 | regulation of cold-induced thermogenesis | 0.000233 | 16 |
| BP | GO:0060411 | cardiac septum morphogenesis | 0.000237 | 11 |
| BP | GO:0072088 | nephron epithelium morphogenesis | 0.000237 | 11 |
| BP | GO:0007043 | cell-cell junction assembly | 0.000237 | 15 |
| BP | GO:0034754 | cellular hormone metabolic process | 0.000237 | 15 |
| BP | GO:0001659 | temperature homeostasis | 0.000244 | 18 |
| BP | GO:0032401 | establishment of melanosome localization | 0.000245 | 6 |
| BP | GO:0051904 | pigment granule transport | 0.000245 | 6 |
| BP | GO:0097205 | renal filtration | 0.000245 | 6 |
| BP | GO:0006509 | membrane protein ectodomain proteolysis | 0.000246 | 8 |
| BP | GO:0021700 | developmental maturation | 0.000249 | 25 |
| BP | GO:0035150 | regulation of tube size | 0.000252 | 16 |
| BP | GO:1901342 | regulation of vasculature development | 0.000262 | 33 |
| BP | GO:0071222 | cellular response to lipopolysaccharide | 0.000269 | 20 |
| BP | GO:0032091 | negative regulation of protein binding | 0.000271 | 13 |
| BP | GO:0030901 | midbrain development | 0.000275 | 12 |
| BP | GO:0046849 | bone remodeling | 0.000275 | 12 |
| BP | GO:0046661 | male sex differentiation | 0.00028 | 17 |
| BP | GO:0007548 | sex differentiation | 0.000285 | 24 |
| BP | GO:0050679 | positive regulation of epithelial cell proliferation | 0.000287 | 20 |
| BP | GO:0045429 | positive regulation of nitric oxide biosynthetic process | 0.000292 | 8 |
| BP | GO:0051353 | positive regulation of oxidoreductase activity | 0.000295 | 9 |
| BP | GO:0090132 | epithelium migration | 0.000297 | 29 |
| BP | GO:0061333 | renal tubule morphogenesis | 0.000299 | 11 |
| BP | GO:0072028 | nephron morphogenesis | 0.000299 | 11 |
| BP | GO:0010837 | regulation of keratinocyte proliferation | 0.0003 | 7 |
| BP | GO:0003158 | endothelium development | 0.000305 | 15 |
| BP | GO:0072329 | monocarboxylic acid catabolic process | 0.000305 | 15 |
| BP | GO:2000116 | regulation of cysteine-type endopeptidase activity | 0.00031 | 22 |
| BP | GO:0001667 | ameboidal-type cell migration | 0.000314 | 35 |
| BP | GO:0051905 | establishment of pigment granule localization | 0.000315 | 6 |
| BP | GO:0046651 | lymphocyte proliferation | 0.000318 | 24 |
| BP | GO:0010975 | regulation of neuron projection development | 0.000325 | 37 |
| BP | GO:0019372 | lipoxygenase pathway | 0.000327 | 5 |
| BP | GO:0070293 | renal absorption | 0.000327 | 5 |
| BP | GO:1905153 | regulation of membrane invagination | 0.000327 | 5 |
| BP | GO:0030193 | regulation of blood coagulation | 0.000335 | 11 |
| BP | GO:0001952 | regulation of cell-matrix adhesion | 0.000335 | 14 |
| BP | GO:0035850 | epithelial cell differentiation involved in kidney development | 0.000344 | 8 |
| BP | GO:0046189 | phenol-containing compound biosynthetic process | 0.000344 | 8 |
| BP | GO:0060412 | ventricular septum morphogenesis | 0.000344 | 8 |
| BP | GO:1904407 | positive regulation of nitric oxide metabolic process | 0.000344 | 8 |
| BP | GO:0014065 | phosphatidylinositol 3-kinase signaling | 0.000345 | 16 |
| BP | GO:0048662 | negative regulation of smooth muscle cell proliferation | 0.00035 | 10 |
| BP | GO:0032943 | mononuclear cell proliferation | 0.000354 | 24 |
| BP | GO:0016486 | peptide hormone processing | 0.000364 | 7 |
| BP | GO:0140448 | signaling receptor ligand precursor processing | 0.000364 | 7 |
| BP | GO:0006721 | terpenoid metabolic process | 0.000365 | 14 |
| BP | GO:0016049 | cell growth | 0.000367 | 36 |
| BP | GO:0043270 | positive regulation of ion transport | 0.000373 | 24 |
| BP | GO:1900046 | regulation of hemostasis | 0.000374 | 11 |
| BP | GO:0033273 | response to vitamin | 0.000375 | 12 |
| BP | GO:0003007 | heart morphogenesis | 0.000383 | 23 |
| BP | GO:0007411 | axon guidance | 0.000393 | 24 |
| BP | GO:0051341 | regulation of oxidoreductase activity | 0.000395 | 13 |
| BP | GO:0032400 | melanosome localization | 0.000401 | 6 |
| BP | GO:0045662 | negative regulation of myoblast differentiation | 0.000401 | 6 |
| BP | GO:0002685 | regulation of leukocyte migration | 0.000411 | 19 |
| BP | GO:0060993 | kidney morphogenesis | 0.000414 | 12 |
| BP | GO:0097485 | neuron projection guidance | 0.000414 | 24 |
| BP | GO:0030111 | regulation of Wnt signaling pathway | 0.000428 | 29 |
| BP | GO:0001893 | maternal placenta development | 0.00044 | 7 |
| BP | GO:0110111 | negative regulation of animal organ morphogenesis | 0.00044 | 7 |
| BP | GO:1905332 | positive regulation of morphogenesis of an epithelium | 0.00044 | 7 |
| BP | GO:0048511 | rhythmic process | 0.000442 | 25 |
| BP | GO:0050766 | positive regulation of phagocytosis | 0.000446 | 10 |
| BP | GO:0071300 | cellular response to retinoic acid | 0.000446 | 10 |
| BP | GO:0030540 | female genitalia development | 0.000448 | 5 |
| BP | GO:1905331 | negative regulation of morphogenesis of an epithelium | 0.000448 | 5 |
| BP | GO:0015850 | organic hydroxy compound transport | 0.000451 | 23 |
| BP | GO:0060485 | mesenchyme development | 0.00046 | 24 |
| BP | GO:0010739 | positive regulation of protein kinase A signaling | 0.000465 | 4 |
| BP | GO:0097278 | complement-dependent cytotoxicity | 0.000465 | 4 |
| BP | GO:1900426 | positive regulation of defense response to bacterium | 0.000465 | 4 |
| BP | GO:0071621 | granulocyte chemotaxis | 0.000471 | 14 |
| BP | GO:0010883 | regulation of lipid storage | 0.000472 | 8 |
| BP | GO:0019731 | antibacterial humoral response | 0.000472 | 8 |
| BP | GO:0001906 | cell killing | 0.000497 | 17 |
| BP | GO:0050729 | positive regulation of inflammatory response | 0.000501 | 16 |
| BP | GO:0051875 | pigment granule localization | 0.000503 | 6 |
| BP | GO:0060669 | embryonic placenta morphogenesis | 0.000503 | 6 |
| BP | GO:0071711 | basement membrane organization | 0.000503 | 6 |
| BP | GO:1900120 | regulation of receptor binding | 0.000503 | 6 |
| BP | GO:0071383 | cellular response to steroid hormone stimulus | 0.000516 | 22 |
| BP | GO:0016101 | diterpenoid metabolic process | 0.000517 | 13 |
| BP | GO:1903532 | positive regulation of secretion by cell | 0.000526 | 31 |
| BP | GO:0000302 | response to reactive oxygen species | 0.000526 | 21 |
| BP | GO:0016042 | lipid catabolic process | 0.000531 | 27 |
| BP | GO:0002700 | regulation of production of molecular mediator of immune response | 0.000532 | 15 |
| BP | GO:0006720 | isoprenoid metabolic process | 0.000532 | 15 |
| BP | GO:0007586 | digestion | 0.000532 | 15 |
| BP | GO:1990845 | adaptive thermogenesis | 0.000538 | 16 |
| BP | GO:0021537 | telencephalon development | 0.000545 | 22 |
| BP | GO:0030574 | collagen catabolic process | 0.000549 | 8 |
| BP | GO:0098868 | bone growth | 0.000549 | 8 |
| BP | GO:0007044 | cell-substrate junction assembly | 0.000554 | 12 |
| BP | GO:0120162 | positive regulation of cold-induced thermogenesis | 0.000554 | 12 |
| BP | GO:0150115 | cell-substrate junction organization | 0.000554 | 12 |
| BP | GO:1990868 | response to chemokine | 0.000554 | 12 |
| BP | GO:1990869 | cellular response to chemokine | 0.000554 | 12 |
| BP | GO:0045471 | response to ethanol | 0.000555 | 14 |
| BP | GO:0010631 | epithelial cell migration | 0.000572 | 28 |
| BP | GO:0043154 | negative regulation of cysteine-type endopeptidase activity involved in apoptotic process | 0.000573 | 11 |
| BP | GO:0050818 | regulation of coagulation | 0.000573 | 11 |
| BP | GO:0042737 | drug catabolic process | 0.000574 | 15 |
| BP | GO:0045071 | negative regulation of viral genome replication | 0.000581 | 9 |
| BP | GO:0097755 | positive regulation of blood vessel diameter | 0.000581 | 9 |
| BP | GO:0120178 | steroid hormone biosynthetic process | 0.000581 | 9 |
| BP | GO:0032970 | regulation of actin filament-based process | 0.000591 | 30 |
| BP | GO:0045683 | negative regulation of epidermis development | 0.0006 | 5 |
| BP | GO:0045667 | regulation of osteoblast differentiation | 0.000602 | 14 |
| BP | GO:1905952 | regulation of lipid localization | 0.00062 | 16 |
| BP | GO:0034405 | response to fluid shear stress | 0.000628 | 7 |
| BP | GO:0051651 | maintenance of location in cell | 0.000632 | 20 |
| BP | GO:0030850 | prostate gland development | 0.000636 | 8 |
| BP | GO:0046850 | regulation of bone remodeling | 0.000636 | 8 |
| BP | GO:0007159 | leukocyte cell-cell adhesion | 0.000638 | 27 |
| BP | GO:0060537 | muscle tissue development | 0.000646 | 31 |
| BP | GO:0071216 | cellular response to biotic stimulus | 0.000658 | 21 |
| BP | GO:0044282 | small molecule catabolic process | 0.000669 | 33 |
| BP | GO:0006959 | humoral immune response | 0.000682 | 28 |
| BP | GO:0045927 | positive regulation of growth | 0.000685 | 23 |
| BP | GO:1903037 | regulation of leukocyte cell-cell adhesion | 0.000686 | 25 |
| BP | GO:0050727 | regulation of inflammatory response | 0.000692 | 29 |
| BP | GO:0071496 | cellular response to external stimulus | 0.000698 | 27 |
| BP | GO:0048013 | ephrin receptor signaling pathway | 0.000701 | 11 |
| BP | GO:0032945 | negative regulation of mononuclear cell proliferation | 0.000704 | 10 |
| BP | GO:0050672 | negative regulation of lymphocyte proliferation | 0.000704 | 10 |
| BP | GO:0032060 | bleb assembly | 0.000708 | 4 |
| BP | GO:0032351 | negative regulation of hormone metabolic process | 0.000708 | 4 |
| BP | GO:0070254 | mucus secretion | 0.000708 | 4 |
| BP | GO:0006690 | icosanoid metabolic process | 0.000729 | 13 |
| BP | GO:0003254 | regulation of membrane depolarization | 0.000733 | 8 |
| BP | GO:0048546 | digestive tract morphogenesis | 0.000733 | 8 |
| BP | GO:0048009 | insulin-like growth factor receptor signaling pathway | 0.000743 | 7 |
| BP | GO:0045604 | regulation of epidermal cell differentiation | 0.000747 | 9 |
| BP | GO:0071384 | cellular response to corticosteroid stimulus | 0.000747 | 9 |
| BP | GO:0070663 | regulation of leukocyte proliferation | 0.00075 | 20 |
| BP | GO:0009314 | response to radiation | 0.000751 | 33 |
| BP | GO:0001991 | regulation of systemic arterial blood pressure by circulatory renin-angiotensin | 0.000787 | 5 |
| BP | GO:0033189 | response to vitamin A | 0.000787 | 5 |
| BP | GO:0051546 | keratinocyte migration | 0.000787 | 5 |
| BP | GO:1902644 | tertiary alcohol metabolic process | 0.000787 | 5 |
| BP | GO:2000696 | regulation of epithelial cell differentiation involved in kidney development | 0.000787 | 5 |
| BP | GO:0046660 | female sex differentiation | 0.000792 | 13 |
| BP | GO:1903426 | regulation of reactive oxygen species biosynthetic process | 0.000799 | 12 |
| BP | GO:0006979 | response to oxidative stress | 0.000841 | 33 |
| BP | GO:0060425 | lung morphogenesis | 0.000842 | 8 |
| BP | GO:0050670 | regulation of lymphocyte proliferation | 0.000852 | 19 |
| BP | GO:0070098 | chemokine-mediated signaling pathway | 0.000853 | 11 |
| BP | GO:0030148 | sphingolipid biosynthetic process | 0.000873 | 12 |
| BP | GO:0008207 | C21-steroid hormone metabolic process | 0.000874 | 7 |
| BP | GO:0003281 | ventricular septum development | 0.000874 | 10 |
| BP | GO:0055074 | calcium ion homeostasis | 0.000893 | 34 |
| BP | GO:0032944 | regulation of mononuclear cell proliferation | 0.000903 | 19 |
| BP | GO:0051146 | striated muscle cell differentiation | 0.00092 | 24 |
| BP | GO:0006936 | muscle contraction | 0.00092 | 28 |
| BP | GO:0018108 | peptidyl-tyrosine phosphorylation | 0.00092 | 28 |
| BP | GO:0003012 | muscle system process | 0.000926 | 34 |
| BP | GO:0044550 | secondary metabolite biosynthetic process | 0.000936 | 6 |
| BP | GO:0046686 | response to cadmium ion | 0.000948 | 9 |
| BP | GO:0018958 | phenol-containing compound metabolic process | 0.000952 | 12 |
| BP | GO:0042552 | myelination | 0.000957 | 14 |
| BP | GO:0001954 | positive regulation of cell-matrix adhesion | 0.000964 | 8 |
| BP | GO:0019369 | arachidonic acid metabolic process | 0.000964 | 8 |
| BP | GO:0007015 | actin filament organization | 0.000965 | 30 |
| BP | GO:0015718 | monocarboxylic acid transport | 0.000998 | 16 |
| BP | GO:0045765 | regulation of angiogenesis | 0.001005 | 29 |
| BP | GO:0072503 | cellular divalent inorganic cation homeostasis | 0.001011 | 35 |
| BP | GO:0033197 | response to vitamin E | 0.001028 | 4 |
| BP | GO:0060670 | branching involved in labyrinthine layer morphogenesis | 0.001028 | 4 |
| BP | GO:1904181 | positive regulation of membrane depolarization | 0.001028 | 4 |
| BP | GO:0018212 | peptidyl-tyrosine modification | 0.001043 | 28 |
| BP | GO:0045600 | positive regulation of fat cell differentiation | 0.001064 | 9 |
| BP | GO:0045669 | positive regulation of osteoblast differentiation | 0.001064 | 9 |
| BP | GO:1901653 | cellular response to peptide | 0.001089 | 29 |
| BP | GO:0031529 | ruffle organization | 0.001099 | 8 |
| BP | GO:0007272 | ensheathment of neurons | 0.001109 | 14 |
| BP | GO:0008366 | axon ensheathment | 0.001109 | 14 |
| BP | GO:0002828 | regulation of type 2 immune response | 0.001131 | 6 |
| BP | GO:0010743 | regulation of macrophage derived foam cell differentiation | 0.001131 | 6 |
| BP | GO:0050680 | negative regulation of epithelial cell proliferation | 0.001137 | 16 |
| BP | GO:0070664 | negative regulation of leukocyte proliferation | 0.00119 | 10 |
| BP | GO:0045833 | negative regulation of lipid metabolic process | 0.001237 | 11 |
| BP | GO:0030195 | negative regulation of blood coagulation | 0.001249 | 8 |
| BP | GO:0031638 | zymogen activation | 0.001249 | 8 |
| BP | GO:0045661 | regulation of myoblast differentiation | 0.001249 | 8 |
| BP | GO:0043281 | regulation of cysteine-type endopeptidase activity involved in apoptotic process | 0.001263 | 19 |
| BP | GO:0070372 | regulation of ERK1 and ERK2 cascade | 0.001271 | 24 |
| BP | GO:0035357 | peroxisome proliferator activated receptor signaling pathway | 0.001288 | 5 |
| BP | GO:0003151 | outflow tract morphogenesis | 0.001314 | 10 |
| BP | GO:0007596 | blood coagulation | 0.001318 | 26 |
| BP | GO:0014706 | striated muscle tissue development | 0.001327 | 29 |
| BP | GO:0019748 | secondary metabolic process | 0.001331 | 9 |
| BP | GO:0001909 | leukocyte mediated cytotoxicity | 0.001333 | 12 |
| BP | GO:0033687 | osteoblast proliferation | 0.001354 | 6 |
| BP | GO:0034694 | response to prostaglandin | 0.001354 | 6 |
| BP | GO:0051955 | regulation of amino acid transport | 0.001354 | 6 |
| BP | GO:0007204 | positive regulation of cytosolic calcium ion concentration | 0.001358 | 25 |
| BP | GO:1905477 | positive regulation of protein localization to membrane | 0.001375 | 13 |
| BP | GO:0034614 | cellular response to reactive oxygen species | 0.001375 | 16 |
| BP | GO:0007389 | pattern specification process | 0.001378 | 32 |
| BP | GO:0042098 | T cell proliferation | 0.00138 | 17 |
| BP | GO:1900047 | negative regulation of hemostasis | 0.001416 | 8 |
| BP | GO:0051604 | protein maturation | 0.001417 | 23 |
| BP | GO:0006705 | mineralocorticoid biosynthetic process | 0.001437 | 4 |
| BP | GO:0008212 | mineralocorticoid metabolic process | 0.001437 | 4 |
| BP | GO:0010839 | negative regulation of keratinocyte proliferation | 0.001437 | 4 |
| BP | GO:0050872 | white fat cell differentiation | 0.001437 | 4 |
| BP | GO:0009062 | fatty acid catabolic process | 0.001445 | 12 |
| BP | GO:0008584 | male gonad development | 0.001474 | 14 |
| BP | GO:0030308 | negative regulation of cell growth | 0.001552 | 17 |
| BP | GO:0071772 | response to BMP | 0.001556 | 16 |
| BP | GO:0071773 | cellular response to BMP stimulus | 0.001556 | 16 |
| BP | GO:0021761 | limbic system development | 0.001566 | 12 |
| BP | GO:0046546 | development of primary male sexual characteristics | 0.001579 | 14 |
| BP | GO:0021762 | substantia nigra development | 0.001591 | 7 |
| BP | GO:0034109 | homotypic cell-cell adhesion | 0.001593 | 10 |
| BP | GO:0019218 | regulation of steroid metabolic process | 0.001595 | 13 |
| BP | GO:0045069 | regulation of viral genome replication | 0.001611 | 11 |
| BP | GO:0003094 | glomerular filtration | 0.001611 | 5 |
| BP | GO:0022010 | central nervous system myelination | 0.001611 | 5 |
| BP | GO:0032291 | axon ensheathment in central nervous system | 0.001611 | 5 |
| BP | GO:0071379 | cellular response to prostaglandin stimulus | 0.001611 | 5 |
| BP | GO:0007599 | hemostasis | 0.001624 | 26 |
| BP | GO:0001666 | response to hypoxia | 0.001627 | 27 |
| BP | GO:0050918 | positive chemotaxis | 0.001649 | 9 |
| BP | GO:0031669 | cellular response to nutrient levels | 0.001668 | 20 |
| BP | GO:0050817 | coagulation | 0.001692 | 26 |
| BP | GO:1904062 | regulation of cation transmembrane transport | 0.001692 | 26 |
| BP | GO:0002573 | myeloid leukocyte differentiation | 0.001705 | 18 |
| BP | GO:0032642 | regulation of chemokine production | 0.00175 | 10 |
| BP | GO:1905954 | positive regulation of lipid localization | 0.00175 | 10 |
| BP | GO:0022409 | positive regulation of cell-cell adhesion | 0.00175 | 21 |
| BP | GO:0097327 | response to antineoplastic agent | 0.001754 | 11 |
| BP | GO:0032956 | regulation of actin cytoskeleton organization | 0.001763 | 26 |
| BP | GO:0050708 | regulation of protein secretion | 0.001785 | 33 |
| BP | GO:0060688 | regulation of morphogenesis of a branching structure | 0.0018 | 8 |
| BP | GO:0001837 | epithelial to mesenchymal transition | 0.001809 | 14 |
| BP | GO:0097530 | granulocyte migration | 0.001809 | 14 |
| BP | GO:0046879 | hormone secretion | 0.00181 | 24 |
| BP | GO:0003416 | endochondral bone growth | 0.001827 | 7 |
| BP | GO:0050730 | regulation of peptidyl-tyrosine phosphorylation | 0.001835 | 21 |
| BP | GO:2000106 | regulation of leukocyte apoptotic process | 0.001919 | 10 |
| BP | GO:0046467 | membrane lipid biosynthetic process | 0.001934 | 14 |
| BP | GO:0090287 | regulation of cellular response to growth factor stimulus | 0.001943 | 23 |
| BP | GO:0072567 | chemokine (C-X-C motif) ligand 2 production | 0.001947 | 4 |
| BP | GO:1900121 | negative regulation of receptor binding | 0.001947 | 4 |
| BP | GO:0046887 | positive regulation of hormone secretion | 0.00198 | 13 |
| BP | GO:0043406 | positive regulation of MAP kinase activity | 0.002018 | 21 |
| BP | GO:0048008 | platelet-derived growth factor receptor signaling pathway | 0.002021 | 8 |
| BP | GO:0050819 | negative regulation of coagulation | 0.002021 | 8 |
| BP | GO:0071398 | cellular response to fatty acid | 0.002021 | 8 |
| BP | GO:0042593 | glucose homeostasis | 0.002034 | 20 |
| BP | GO:0090092 | regulation of transmembrane receptor protein serine/threonine kinase signaling pathway | 0.002034 | 20 |
| BP | GO:0030038 | contractile actin filament bundle assembly | 0.002072 | 11 |
| BP | GO:0043149 | stress fiber assembly | 0.002072 | 11 |
| BP | GO:0050764 | regulation of phagocytosis | 0.002072 | 11 |
| BP | GO:0046165 | alcohol biosynthetic process | 0.002098 | 16 |
| BP | GO:0006874 | cellular calcium ion homeostasis | 0.0021 | 32 |
| BP | GO:0051492 | regulation of stress fiber assembly | 0.002101 | 10 |
| BP | GO:0097756 | negative regulation of blood vessel diameter | 0.002101 | 10 |
| BP | GO:0002687 | positive regulation of leukocyte migration | 0.002124 | 13 |
| BP | GO:0033500 | carbohydrate homeostasis | 0.002135 | 20 |
| BP | GO:0050731 | positive regulation of peptidyl-tyrosine phosphorylation | 0.00218 | 17 |
| BP | GO:0072659 | protein localization to plasma membrane | 0.002215 | 21 |
| BP | GO:0035904 | aorta development | 0.002262 | 8 |
| BP | GO:0071385 | cellular response to glucocorticoid stimulus | 0.002262 | 8 |
| BP | GO:1904589 | regulation of protein import | 0.002262 | 8 |
| BP | GO:0002067 | glandular epithelial cell differentiation | 0.002379 | 7 |
| BP | GO:0002791 | regulation of peptide secretion | 0.002411 | 34 |
| BP | GO:0003002 | regionalization | 0.002423 | 26 |
| BP | GO:0001783 | B cell apoptotic process | 0.002432 | 5 |
| BP | GO:0003433 | chondrocyte development involved in endochondral bone morphogenesis | 0.002432 | 5 |
| BP | GO:0046697 | decidualization | 0.002432 | 5 |
| BP | GO:0036293 | response to decreased oxygen levels | 0.002493 | 27 |
| BP | GO:0042446 | hormone biosynthetic process | 0.002506 | 10 |
| BP | GO:0001885 | endothelial cell development | 0.002526 | 8 |
| BP | GO:0032890 | regulation of organic acid transport | 0.002526 | 8 |
| BP | GO:0033619 | membrane protein proteolysis | 0.002526 | 8 |
| BP | GO:0042130 | negative regulation of T cell proliferation | 0.002526 | 8 |
| BP | GO:0032274 | gonadotropin secretion | 0.002571 | 4 |
| BP | GO:0044406 | adhesion of symbiont to host | 0.002571 | 4 |
| BP | GO:0048569 | post-embryonic animal organ development | 0.002571 | 4 |
| BP | GO:0034765 | regulation of ion transmembrane transport | 0.002574 | 33 |
| BP | GO:0042092 | type 2 immune response | 0.002602 | 6 |
| BP | GO:0010821 | regulation of mitochondrion organization | 0.002637 | 16 |
| BP | GO:0009914 | hormone transport | 0.002638 | 24 |
| BP | GO:0070371 | ERK1 and ERK2 cascade | 0.002638 | 24 |
| BP | GO:0021782 | glial cell development | 0.002659 | 12 |
| BP | GO:0048806 | genitalia development | 0.002699 | 7 |
| BP | GO:0060986 | endocrine hormone secretion | 0.002699 | 7 |
| BP | GO:0006694 | steroid biosynthetic process | 0.002707 | 17 |
| BP | GO:0060350 | endochondral bone morphogenesis | 0.002715 | 9 |
| BP | GO:0002690 | positive regulation of leukocyte chemotaxis | 0.002731 | 10 |
| BP | GO:1905330 | regulation of morphogenesis of an epithelium | 0.002789 | 16 |
| BP | GO:0002367 | cytokine production involved in immune response | 0.002848 | 11 |
| BP | GO:0031069 | hair follicle morphogenesis | 0.002939 | 5 |
| BP | GO:0031639 | plasminogen activation | 0.002939 | 5 |
| BP | GO:0032616 | interleukin-13 production | 0.002939 | 5 |
| BP | GO:0050927 | positive regulation of positive chemotaxis | 0.002939 | 5 |
| BP | GO:0060571 | morphogenesis of an epithelial fold | 0.002939 | 5 |
| BP | GO:0061217 | regulation of mesonephros development | 0.002939 | 5 |
| BP | GO:0072202 | cell differentiation involved in metanephros development | 0.002939 | 5 |
| BP | GO:0071560 | cellular response to transforming growth factor beta stimulus | 0.00297 | 20 |
| BP | GO:0034103 | regulation of tissue remodeling | 0.002972 | 10 |
| BP | GO:1901616 | organic hydroxy compound catabolic process | 0.002983 | 9 |
| BP | GO:0010742 | macrophage derived foam cell differentiation | 0.003017 | 6 |
| BP | GO:0042554 | superoxide anion generation | 0.003017 | 6 |
| BP | GO:0060428 | lung epithelium development | 0.003017 | 6 |
| BP | GO:0090077 | foam cell differentiation | 0.003017 | 6 |
| BP | GO:0090322 | regulation of superoxide metabolic process | 0.003017 | 6 |
| BP | GO:0097242 | amyloid-beta clearance | 0.003017 | 6 |
| BP | GO:0060324 | face development | 0.003051 | 7 |
| BP | GO:0070482 | response to oxygen levels | 0.003053 | 28 |
| BP | GO:0007229 | integrin-mediated signaling pathway | 0.003074 | 11 |
| BP | GO:0003170 | heart valve development | 0.003125 | 8 |
| BP | GO:0006081 | cellular aldehyde metabolic process | 0.003125 | 8 |
| BP | GO:0046513 | ceramide biosynthetic process | 0.003125 | 8 |
| BP | GO:0031668 | cellular response to extracellular stimulus | 0.003176 | 21 |
| BP | GO:0050714 | positive regulation of protein secretion | 0.003176 | 21 |
| BP | GO:0032355 | response to estradiol | 0.00318 | 13 |
| BP | GO:0090288 | negative regulation of cellular response to growth factor stimulus | 0.003187 | 15 |
| BP | GO:0016331 | morphogenesis of embryonic epithelium | 0.003214 | 14 |
| BP | GO:0060828 | regulation of canonical Wnt signaling pathway | 0.003215 | 22 |
| BP | GO:0050770 | regulation of axonogenesis | 0.003287 | 16 |
| BP | GO:0008630 | intrinsic apoptotic signaling pathway in response to DNA damage | 0.003315 | 11 |
| BP | GO:0030593 | neutrophil chemotaxis | 0.003315 | 11 |
| BP | GO:0002830 | positive regulation of type 2 immune response | 0.003319 | 4 |
| BP | GO:0006837 | serotonin transport | 0.003319 | 4 |
| BP | GO:0006957 | complement activation, alternative pathway | 0.003319 | 4 |
| BP | GO:0010566 | regulation of ketone biosynthetic process | 0.003319 | 4 |
| BP | GO:0048385 | regulation of retinoic acid receptor signaling pathway | 0.003319 | 4 |
| BP | GO:0048820 | hair follicle maturation | 0.003319 | 4 |
| BP | GO:0060572 | morphogenesis of an epithelial bud | 0.003319 | 4 |
| BP | GO:0008406 | gonad development | 0.00334 | 18 |
| BP | GO:0044242 | cellular lipid catabolic process | 0.00334 | 18 |
| BP | GO:0030857 | negative regulation of epithelial cell differentiation | 0.003438 | 7 |
| BP | GO:0002683 | negative regulation of immune system process | 0.003443 | 30 |
| BP | GO:0032835 | glomerulus development | 0.003463 | 8 |
| BP | GO:1901862 | negative regulation of muscle tissue development | 0.003463 | 8 |
| BP | GO:0051650 | establishment of vesicle localization | 0.003469 | 16 |
| BP | GO:0060560 | developmental growth involved in morphogenesis | 0.003469 | 19 |
| BP | GO:0002793 | positive regulation of peptide secretion | 0.003495 | 22 |
| BP | GO:0019722 | calcium-mediated signaling | 0.003507 | 18 |
| BP | GO:0030318 | melanocyte differentiation | 0.003519 | 5 |
| BP | GO:0042044 | fluid transport | 0.003519 | 5 |
| BP | GO:0043567 | regulation of insulin-like growth factor receptor signaling pathway | 0.003519 | 5 |
| BP | GO:0050926 | regulation of positive chemotaxis | 0.003519 | 5 |
| BP | GO:0060706 | cell differentiation involved in embryonic placenta development | 0.003519 | 5 |
| BP | GO:0060740 | prostate gland epithelium morphogenesis | 0.003519 | 5 |
| BP | GO:0072089 | stem cell proliferation | 0.003521 | 12 |
| BP | GO:2000027 | regulation of animal organ morphogenesis | 0.00356 | 20 |
| BP | GO:0007156 | homophilic cell adhesion via plasma membrane adhesion molecules | 0.00357 | 15 |
| BP | GO:0042310 | vasoconstriction | 0.003579 | 9 |
| BP | GO:1903169 | regulation of calcium ion transmembrane transport | 0.003624 | 14 |
| BP | GO:0097193 | intrinsic apoptotic signaling pathway | 0.003643 | 22 |
| BP | GO:0051924 | regulation of calcium ion transport | 0.003722 | 20 |
| BP | GO:0010811 | positive regulation of cell-substrate adhesion | 0.003768 | 12 |
| BP | GO:0070252 | actin-mediated cell contraction | 0.003768 | 12 |
| BP | GO:0010469 | regulation of signaling receptor activity | 0.003775 | 15 |
| BP | GO:0010718 | positive regulation of epithelial to mesenchymal transition | 0.00386 | 7 |
| BP | GO:0033059 | cellular pigmentation | 0.00386 | 7 |
| BP | GO:0048762 | mesenchymal cell differentiation | 0.003862 | 18 |
| BP | GO:0071559 | response to transforming growth factor beta | 0.003889 | 20 |
| BP | GO:0006809 | nitric oxide biosynthetic process | 0.003911 | 9 |
| BP | GO:0051702 | interaction with symbiont | 0.003911 | 9 |
| BP | GO:0019932 | second-messenger-mediated signaling | 0.003928 | 30 |
| BP | GO:0071356 | cellular response to tumor necrosis factor | 0.003955 | 22 |
| BP | GO:0032350 | regulation of hormone metabolic process | 0.003993 | 6 |
| BP | GO:0060416 | response to growth hormone | 0.003993 | 6 |
| BP | GO:1905475 | regulation of protein localization to membrane | 0.004064 | 16 |
| BP | GO:0034764 | positive regulation of transmembrane transport | 0.00408 | 17 |
| BP | GO:0048799 | animal organ maturation | 0.004177 | 5 |
| BP | GO:1901623 | regulation of lymphocyte chemotaxis | 0.004177 | 5 |
| BP | GO:0010744 | positive regulation of macrophage derived foam cell differentiation | 0.004202 | 4 |
| BP | GO:0061318 | renal filtration cell differentiation | 0.004202 | 4 |
| BP | GO:0072112 | glomerular visceral epithelial cell differentiation | 0.004202 | 4 |
| BP | GO:0050922 | negative regulation of chemotaxis | 0.004224 | 8 |
| BP | GO:0071248 | cellular response to metal ion | 0.00428 | 16 |
| BP | GO:0051928 | positive regulation of calcium ion transport | 0.004304 | 12 |
| BP | GO:0007566 | embryo implantation | 0.004321 | 7 |
| BP | GO:0045599 | negative regulation of fat cell differentiation | 0.004321 | 7 |
| BP | GO:0050663 | cytokine secretion | 0.00436 | 19 |
| BP | GO:0042742 | defense response to bacterium | 0.004387 | 24 |
| BP | GO:0045137 | development of primary sexual characteristics | 0.004449 | 18 |
| BP | GO:0071214 | cellular response to abiotic stimulus | 0.004555 | 24 |
| BP | GO:0104004 | cellular response to environmental stimulus | 0.004555 | 24 |
| BP | GO:0033574 | response to testosterone | 0.00456 | 6 |
| BP | GO:2001233 | regulation of apoptotic signaling pathway | 0.004616 | 28 |
| BP | GO:0062197 | cellular response to chemical stress | 0.004645 | 25 |
| BP | GO:0006026 | aminoglycan catabolic process | 0.00465 | 8 |
| BP | GO:0032768 | regulation of monooxygenase activity | 0.00465 | 8 |
| BP | GO:0006643 | membrane lipid metabolic process | 0.004724 | 17 |
| BP | GO:0009416 | response to light stimulus | 0.004792 | 23 |
| BP | GO:0050863 | regulation of T cell activation | 0.004792 | 23 |
| BP | GO:0003179 | heart valve morphogenesis | 0.004822 | 7 |
| BP | GO:0055078 | sodium ion homeostasis | 0.004822 | 7 |
| BP | GO:0008045 | motor neuron axon guidance | 0.004918 | 5 |
| BP | GO:0008209 | androgen metabolic process | 0.004918 | 5 |
| BP | GO:0033688 | regulation of osteoblast proliferation | 0.004918 | 5 |
| BP | GO:0034698 | response to gonadotropin | 0.004918 | 5 |
| BP | GO:0035116 | embryonic hindlimb morphogenesis | 0.004918 | 5 |
| BP | GO:0042634 | regulation of hair cycle | 0.004918 | 5 |
| BP | GO:0042730 | fibrinolysis | 0.004918 | 5 |
| BP | GO:0007623 | circadian rhythm | 0.004956 | 17 |
| BP | GO:0042594 | response to starvation | 0.004984 | 16 |
| BP | GO:0071478 | cellular response to radiation | 0.004984 | 16 |
| BP | GO:1903522 | regulation of blood circulation | 0.005026 | 22 |
| BP | GO:0051495 | positive regulation of cytoskeleton organization | 0.005108 | 18 |
| BP | GO:0045428 | regulation of nitric oxide biosynthetic process | 0.005108 | 8 |
| BP | GO:0002702 | positive regulation of production of molecular mediator of immune response | 0.005169 | 10 |
| BP | GO:0044070 | regulation of anion transport | 0.005169 | 10 |
| BP | GO:0110020 | regulation of actomyosin structure organization | 0.005169 | 10 |
| BP | GO:0001709 | cell fate determination | 0.005184 | 6 |
| BP | GO:0042036 | negative regulation of cytokine biosynthetic process | 0.005184 | 6 |
| BP | GO:0050832 | defense response to fungus | 0.005184 | 6 |
| BP | GO:0051281 | positive regulation of release of sequestered calcium ion into cytosol | 0.005184 | 6 |
| BP | GO:0072210 | metanephric nephron development | 0.005184 | 6 |
| BP | GO:0048706 | embryonic skeletal system development | 0.005222 | 12 |
| BP | GO:0003414 | chondrocyte morphogenesis involved in endochondral bone morphogenesis | 0.005231 | 4 |
| BP | GO:0003429 | growth plate cartilage chondrocyte morphogenesis | 0.005231 | 4 |
| BP | GO:0044320 | cellular response to leptin stimulus | 0.005231 | 4 |
| BP | GO:0072311 | glomerular epithelial cell differentiation | 0.005231 | 4 |
| BP | GO:0090171 | chondrocyte morphogenesis | 0.005231 | 4 |
| BP | GO:0090196 | regulation of chemokine secretion | 0.005231 | 4 |
| BP | GO:0046883 | regulation of hormone secretion | 0.005249 | 20 |
| BP | GO:0060563 | neuroepithelial cell differentiation | 0.005366 | 7 |
| BP | GO:0070228 | regulation of lymphocyte apoptotic process | 0.005366 | 7 |
| BP | GO:0006665 | sphingolipid metabolic process | 0.005408 | 14 |
| BP | GO:0048708 | astrocyte differentiation | 0.005482 | 9 |
| BP | GO:0110110 | positive regulation of animal organ morphogenesis | 0.005482 | 9 |
| BP | GO:0051897 | positive regulation of protein kinase B signaling | 0.005497 | 15 |
| BP | GO:0035270 | endocrine system development | 0.005561 | 12 |
| BP | GO:0050909 | sensory perception of taste | 0.005599 | 8 |
| BP | GO:0033032 | regulation of myeloid cell apoptotic process | 0.005748 | 5 |
| BP | GO:0060142 | regulation of syncytium formation by plasma membrane fusion | 0.005748 | 5 |
| BP | GO:0060441 | epithelial tube branching involved in lung morphogenesis | 0.005748 | 5 |
| BP | GO:0060536 | cartilage morphogenesis | 0.005748 | 5 |
| BP | GO:1900027 | regulation of ruffle assembly | 0.005748 | 5 |
| BP | GO:0048469 | cell maturation | 0.005788 | 15 |
| BP | GO:0007520 | myoblast fusion | 0.005868 | 6 |
| BP | GO:0045616 | regulation of keratinocyte differentiation | 0.005868 | 6 |
| BP | GO:0048286 | lung alveolus development | 0.005868 | 6 |
| BP | GO:0061028 | establishment of endothelial barrier | 0.005868 | 6 |
| BP | GO:0085029 | extracellular matrix assembly | 0.005868 | 6 |
| BP | GO:0097178 | ruffle assembly | 0.005868 | 6 |
| BP | GO:0140353 | lipid export from cell | 0.005868 | 6 |
| BP | GO:0046209 | nitric oxide metabolic process | 0.005943 | 9 |
| BP | GO:0051279 | regulation of release of sequestered calcium ion into cytosol | 0.005943 | 9 |
| BP | GO:0051480 | regulation of cytosolic calcium ion concentration | 0.00595 | 25 |
| BP | GO:0010524 | positive regulation of calcium ion transport into cytosol | 0.005954 | 7 |
| BP | GO:1904645 | response to amyloid-beta | 0.005954 | 7 |
| BP | GO:0035690 | cellular response to drug | 0.005965 | 29 |
| BP | GO:0032231 | regulation of actin filament bundle assembly | 0.005986 | 10 |
| BP | GO:0034599 | cellular response to oxidative stress | 0.006093 | 22 |
| BP | GO:0019915 | lipid storage | 0.006126 | 8 |
| BP | GO:0042391 | regulation of membrane potential | 0.006154 | 29 |
| BP | GO:0010876 | lipid localization | 0.006188 | 28 |
| BP | GO:1903038 | negative regulation of leukocyte cell-cell adhesion | 0.006289 | 12 |
| BP | GO:2001235 | positive regulation of apoptotic signaling pathway | 0.006407 | 15 |
| BP | GO:0002689 | negative regulation of leukocyte chemotaxis | 0.006417 | 4 |
| BP | GO:0003159 | morphogenesis of an endothelium | 0.006417 | 4 |
| BP | GO:0003422 | growth plate cartilage morphogenesis | 0.006417 | 4 |
| BP | GO:0006833 | water transport | 0.006417 | 4 |
| BP | GO:0015669 | gas transport | 0.006417 | 4 |
| BP | GO:0060231 | mesenchymal to epithelial transition | 0.006417 | 4 |
| BP | GO:0061154 | endothelial tube morphogenesis | 0.006417 | 4 |
| BP | GO:0045807 | positive regulation of endocytosis | 0.006431 | 10 |
| BP | GO:0006970 | response to osmotic stress | 0.006432 | 9 |
| BP | GO:0032275 | luteinizing hormone secretion | 0.006457 | 3 |
| BP | GO:0032341 | aldosterone metabolic process | 0.006457 | 3 |
| BP | GO:0032342 | aldosterone biosynthetic process | 0.006457 | 3 |
| BP | GO:0032353 | negative regulation of hormone biosynthetic process | 0.006457 | 3 |
| BP | GO:0034650 | cortisol metabolic process | 0.006457 | 3 |
| BP | GO:0042758 | long-chain fatty acid catabolic process | 0.006457 | 3 |
| BP | GO:0045741 | positive regulation of epidermal growth factor-activated receptor activity | 0.006457 | 3 |
| BP | GO:0046322 | negative regulation of fatty acid oxidation | 0.006457 | 3 |
| BP | GO:0051901 | positive regulation of mitochondrial depolarization | 0.006457 | 3 |
| BP | GO:0061140 | lung secretory cell differentiation | 0.006457 | 3 |
| BP | GO:0070099 | regulation of chemokine-mediated signaling pathway | 0.006457 | 3 |
| BP | GO:0070942 | neutrophil mediated cytotoxicity | 0.006457 | 3 |
| BP | GO:0072015 | glomerular visceral epithelial cell development | 0.006457 | 3 |
| BP | GO:0090030 | regulation of steroid hormone biosynthetic process | 0.006457 | 3 |
| BP | GO:0090557 | establishment of endothelial intestinal barrier | 0.006457 | 3 |
| BP | GO:0098722 | asymmetric stem cell division | 0.006457 | 3 |
| BP | GO:0003229 | ventricular cardiac muscle tissue development | 0.006589 | 7 |
| BP | GO:0042306 | regulation of protein import into nucleus | 0.006589 | 7 |
| BP | GO:0098900 | regulation of action potential | 0.006589 | 7 |
| BP | GO:0015701 | bicarbonate transport | 0.006617 | 6 |
| BP | GO:0017145 | stem cell division | 0.006617 | 6 |
| BP | GO:0031641 | regulation of myelination | 0.006617 | 6 |
| BP | GO:0045446 | endothelial cell differentiation | 0.006654 | 11 |
| BP | GO:0003413 | chondrocyte differentiation involved in endochondral bone morphogenesis | 0.006671 | 5 |
| BP | GO:1904950 | negative regulation of establishment of protein localization | 0.006678 | 16 |
| BP | GO:0015711 | organic anion transport | 0.006689 | 32 |
| BP | GO:0071241 | cellular response to inorganic substance | 0.006857 | 17 |
| BP | GO:0019395 | fatty acid oxidation | 0.006901 | 10 |
| BP | GO:0062014 | negative regulation of small molecule metabolic process | 0.006901 | 10 |
| BP | GO:1901570 | fatty acid derivative biosynthetic process | 0.006901 | 10 |
| BP | GO:0090263 | positive regulation of canonical Wnt signaling pathway | 0.006948 | 13 |
| BP | GO:0002718 | regulation of cytokine production involved in immune response | 0.006952 | 9 |
| BP | GO:0048738 | cardiac muscle tissue development | 0.006959 | 18 |
| BP | GO:0051208 | sequestering of calcium ion | 0.007092 | 12 |
| BP | GO:0000768 | syncytium formation by plasma membrane fusion | 0.007273 | 7 |
| BP | GO:0046148 | pigment biosynthetic process | 0.007273 | 7 |
| BP | GO:0086065 | cell communication involved in cardiac conduction | 0.007273 | 7 |
| BP | GO:0140253 | cell-cell fusion | 0.007273 | 7 |
| BP | GO:0035148 | tube formation | 0.007343 | 13 |
| BP | GO:0015837 | amine transport | 0.007398 | 10 |
| BP | GO:0045746 | negative regulation of Notch signaling pathway | 0.007432 | 6 |
| BP | GO:2001242 | regulation of intrinsic apoptotic signaling pathway | 0.007446 | 14 |
| BP | GO:0002028 | regulation of sodium ion transport | 0.007503 | 9 |
| BP | GO:1903901 | negative regulation of viral life cycle | 0.007503 | 9 |
| BP | GO:2001057 | reactive nitrogen species metabolic process | 0.007503 | 9 |
| BP | GO:0072175 | epithelial tube formation | 0.007522 | 12 |
| BP | GO:0010822 | positive regulation of mitochondrion organization | 0.007561 | 11 |
| BP | GO:0003215 | cardiac right ventricle morphogenesis | 0.007767 | 4 |
| BP | GO:0010738 | regulation of protein kinase A signaling | 0.007767 | 4 |
| BP | GO:0051797 | regulation of hair follicle development | 0.007767 | 4 |
| BP | GO:0090195 | chemokine secretion | 0.007767 | 4 |
| BP | GO:0031349 | positive regulation of defense response | 0.007794 | 26 |
| BP | GO:0016125 | sterol metabolic process | 0.007839 | 14 |
| BP | GO:0032868 | response to insulin | 0.007849 | 20 |
| BP | GO:0034440 | lipid oxidation | 0.007921 | 10 |
| BP | GO:0048525 | negative regulation of viral process | 0.007921 | 10 |
| BP | GO:1904427 | positive regulation of calcium ion transmembrane transport | 0.007932 | 8 |
| CC | GO:0062023 | collagen-containing extracellular matrix | 9.17E-17 | 57 |
| CC | GO:0001533 | cornified envelope | 2.86E-15 | 22 |
| CC | GO:0030057 | desmosome | 6.66E-14 | 14 |
| CC | GO:0005911 | cell-cell junction | 3.21E-12 | 50 |
| CC | GO:0045177 | apical part of cell | 1.02E-09 | 43 |
| CC | GO:0016324 | apical plasma membrane | 5.41E-09 | 37 |
| CC | GO:0016323 | basolateral plasma membrane | 5.73E-07 | 26 |
| CC | GO:0016327 | apicolateral plasma membrane | 3.34E-06 | 7 |
| CC | GO:0009925 | basal plasma membrane | 5.14E-06 | 9 |
| CC | GO:0016328 | lateral plasma membrane | 1.28E-05 | 11 |
| CC | GO:0005788 | endoplasmic reticulum lumen | 1.82E-05 | 29 |
| CC | GO:0043292 | contractile fiber | 2.26E-05 | 24 |
| CC | GO:0045178 | basal part of cell | 2.74E-05 | 10 |
| CC | GO:0070160 | tight junction | 4.97E-05 | 16 |
| CC | GO:0031252 | cell leading edge | 7.81E-05 | 33 |
| CC | GO:0019897 | extrinsic component of plasma membrane | 0.000106 | 18 |
| CC | GO:0005923 | bicellular tight junction | 0.000113 | 15 |
| CC | GO:0030055 | cell-substrate junction | 0.000119 | 33 |
| CC | GO:0031983 | vesicle lumen | 0.000126 | 28 |
| CC | GO:0005882 | intermediate filament | 0.000135 | 21 |
| CC | GO:0019898 | extrinsic component of membrane | 0.000137 | 26 |
| CC | GO:0035580 | specific granule lumen | 0.000156 | 10 |
| CC | GO:0043296 | apical junction complex | 0.000187 | 16 |
| CC | GO:0034774 | secretory granule lumen | 0.000221 | 27 |
| CC | GO:0060205 | cytoplasmic vesicle lumen | 0.00027 | 27 |
| CC | GO:0042470 | melanosome | 0.000299 | 13 |
| CC | GO:0048770 | pigment granule | 0.000299 | 13 |
| CC | GO:0005925 | focal adhesion | 0.000416 | 31 |
| CC | GO:0005916 | fascia adherens | 0.000432 | 4 |
| CC | GO:0045111 | intermediate filament cytoskeleton | 0.00047 | 22 |
| CC | GO:0001726 | ruffle | 0.000525 | 17 |
| CC | GO:0030016 | myofibril | 0.000662 | 20 |
| CC | GO:0042581 | specific granule | 0.000666 | 16 |
| CC | GO:0043034 | costamere | 0.000721 | 5 |
| CC | GO:0005912 | adherens junction | 0.000837 | 10 |
| CC | GO:0031594 | neuromuscular junction | 0.000837 | 10 |
| CC | GO:0070820 | tertiary granule | 0.000871 | 16 |
| CC | GO:0001725 | stress fiber | 0.001297 | 9 |
| CC | GO:0097517 | contractile actin filament bundle | 0.001297 | 9 |
| CC | GO:0030017 | sarcomere | 0.001379 | 18 |
| CC | GO:0033162 | melanosome membrane | 0.001813 | 4 |
| CC | GO:0045009 | chitosome | 0.001813 | 4 |
| CC | GO:0090741 | pigment granule membrane | 0.001813 | 4 |
| CC | GO:0030665 | clathrin-coated vesicle membrane | 0.002109 | 12 |
| CC | GO:0005796 | Golgi lumen | 0.002454 | 11 |
| CC | GO:0032432 | actin filament bundle | 0.002875 | 9 |
| CC | GO:0031253 | cell projection membrane | 0.002961 | 24 |
| CC | GO:0043025 | neuronal cell body | 0.002987 | 33 |
| CC | GO:0043209 | myelin sheath | 0.003087 | 7 |
| CC | GO:0031225 | anchored component of membrane | 0.003337 | 15 |
| CC | GO:0030136 | clathrin-coated vesicle | 0.003556 | 16 |
| CC | GO:0005775 | vacuolar lumen | 0.003727 | 15 |
| CC | GO:0005771 | multivesicular body | 0.003885 | 7 |
| CC | GO:0042599 | lamellar body | 0.003919 | 4 |
| CC | GO:0101002 | ficolin-1-rich granule | 0.003943 | 12 |
| CC | GO:1904813 | ficolin-1-rich granule lumen | 0.003943 | 12 |
| CC | GO:0045121 | membrane raft | 0.003956 | 23 |
| CC | GO:0042641 | actomyosin | 0.004095 | 9 |
| CC | GO:0098857 | membrane microdomain | 0.004111 | 23 |
| CC | GO:0009897 | external side of plasma membrane | 0.004373 | 27 |
| CC | GO:0030139 | endocytic vesicle | 0.005082 | 22 |
| CC | GO:1904724 | tertiary granule lumen | 0.005941 | 7 |
| CC | GO:0030135 | coated vesicle | 0.006049 | 21 |
| CC | GO:0030662 | coated vesicle membrane | 0.00627 | 15 |
| CC | GO:0098589 | membrane region | 0.006408 | 23 |
| CC | GO:0030018 | Z disc | 0.00649 | 12 |
| MF | GO:0005200 | structural constituent of cytoskeleton | 1.11E-08 | 20 |
| MF | GO:0004866 | endopeptidase inhibitor activity | 5.13E-07 | 24 |
| MF | GO:0098632 | cell-cell adhesion mediator activity | 1.00E-06 | 12 |
| MF | GO:0030414 | peptidase inhibitor activity | 1.06E-06 | 24 |
| MF | GO:0061135 | endopeptidase regulator activity | 1.06E-06 | 24 |
| MF | GO:0050839 | cell adhesion molecule binding | 2.42E-06 | 45 |
| MF | GO:0061134 | peptidase regulator activity | 2.86E-06 | 26 |
| MF | GO:0098631 | cell adhesion mediator activity | 6.47E-06 | 12 |
| MF | GO:0005506 | iron ion binding | 8.52E-06 | 20 |
| MF | GO:0016825 | hydrolase activity, acting on acid phosphorus-nitrogen bonds | 1.73E-05 | 22 |
| MF | GO:0017171 | serine hydrolase activity | 1.73E-05 | 22 |
| MF | GO:0004252 | serine-type endopeptidase activity | 1.84E-05 | 20 |
| MF | GO:0019215 | intermediate filament binding | 2.24E-05 | 6 |
| MF | GO:0005201 | extracellular matrix structural constituent | 2.42E-05 | 20 |
| MF | GO:0008236 | serine-type peptidase activity | 3.88E-05 | 21 |
| MF | GO:0045236 | CXCR chemokine receptor binding | 5.44E-05 | 5 |
| MF | GO:0003779 | actin binding | 5.45E-05 | 37 |
| MF | GO:0005518 | collagen binding | 0.000124 | 11 |
| MF | GO:0019199 | transmembrane receptor protein kinase activity | 0.000134 | 12 |
| MF | GO:0016705 | oxidoreductase activity, acting on paired donors, with incorporation or reduction of molecular oxygen | 0.000173 | 18 |
| MF | GO:0004867 | serine-type endopeptidase inhibitor activity | 0.00019 | 13 |
| MF | GO:0004714 | transmembrane receptor protein tyrosine kinase activity | 0.000292 | 10 |
| MF | GO:0004029 | aldehyde dehydrogenase (NAD+) activity | 0.000306 | 5 |
| MF | GO:0001228 | DNA-binding transcription activator activity, RNA polymerase II-specific | 0.000357 | 35 |
| MF | GO:0001216 | DNA-binding transcription activator activity | 0.000372 | 35 |
| MF | GO:0005539 | glycosaminoglycan binding | 0.00038 | 22 |
| MF | GO:0004030 | aldehyde dehydrogenase [NAD(P)+] activity | 0.000429 | 5 |
| MF | GO:0030280 | structural constituent of skin epidermis | 0.000429 | 5 |
| MF | GO:0016903 | oxidoreductase activity, acting on the aldehyde or oxo group of donors | 0.000433 | 8 |
| MF | GO:0046906 | tetrapyrrole binding | 0.000519 | 16 |
| MF | GO:0045296 | cadherin binding | 0.000539 | 28 |
| MF | GO:0005125 | cytokine activity | 0.000557 | 21 |
| MF | GO:0043177 | organic acid binding | 0.000566 | 20 |
| MF | GO:0016620 | oxidoreductase activity, acting on the aldehyde or oxo group of donors, NAD or NADP as acceptor | 0.000625 | 7 |
| MF | GO:0031406 | carboxylic acid binding | 0.000689 | 19 |
| MF | GO:0020037 | heme binding | 0.000714 | 15 |
| MF | GO:0019838 | growth factor binding | 0.000832 | 15 |
| MF | GO:0004857 | enzyme inhibitor activity | 0.000868 | 30 |
| MF | GO:0015250 | water channel activity | 0.000883 | 4 |
| MF | GO:0015643 | toxic substance binding | 0.000883 | 4 |
| MF | GO:0050786 | RAGE receptor binding | 0.000883 | 4 |
| MF | GO:0004175 | endopeptidase activity | 0.000889 | 33 |
| MF | GO:0008201 | heparin binding | 0.001017 | 17 |
| MF | GO:0098641 | cadherin binding involved in cell-cell adhesion | 0.001026 | 5 |
| MF | GO:0004497 | monooxygenase activity | 0.001109 | 12 |
| MF | GO:0030546 | signaling receptor activator activity | 0.001177 | 36 |
| MF | GO:0016709 | oxidoreductase activity, acting on paired donors, with incorporation or reduction of molecular oxygen, NAD(P)H as one donor, and incorporation of one atom of oxygen | 0.001231 | 7 |
| MF | GO:0016614 | oxidoreductase activity, acting on CH-OH group of donors | 0.001238 | 14 |
| MF | GO:0019955 | cytokine binding | 0.001238 | 14 |
| MF | GO:0005372 | water transmembrane transporter activity | 0.00128 | 4 |
| MF | GO:0031994 | insulin-like growth factor I binding | 0.00128 | 4 |
| MF | GO:0045295 | gamma-catenin binding | 0.00128 | 4 |
| MF | GO:0005178 | integrin binding | 0.001665 | 14 |
| MF | GO:0030548 | acetylcholine receptor regulator activity | 0.001786 | 4 |
| MF | GO:0039706 | co-receptor binding | 0.001786 | 4 |
| MF | GO:0086080 | protein binding involved in heterotypic cell-cell adhesion | 0.001786 | 4 |
| MF | GO:0099602 | neurotransmitter receptor regulator activity | 0.001786 | 4 |
| MF | GO:0016616 | oxidoreductase activity, acting on the CH-OH group of donors, NAD or NADP as acceptor | 0.001843 | 13 |
| MF | GO:0048018 | receptor ligand activity | 0.001849 | 35 |
| MF | GO:0042379 | chemokine receptor binding | 0.001992 | 9 |
| MF | GO:0051015 | actin filament binding | 0.002315 | 18 |
| MF | GO:0036041 | long-chain fatty acid binding | 0.002416 | 4 |
| MF | GO:0070330 | aromatase activity | 0.002416 | 4 |
| MF | GO:0000900 | translation repressor activity, mRNA regulatory element binding | 0.003183 | 4 |
| MF | GO:0033218 | amide binding | 0.003258 | 27 |
| MF | GO:0008307 | structural constituent of muscle | 0.003301 | 7 |
| MF | GO:0016229 | steroid dehydrogenase activity | 0.003483 | 6 |
| MF | GO:0002020 | protease binding | 0.003514 | 13 |
| MF | GO:0097110 | scaffold protein binding | 0.003618 | 8 |
| MF | GO:0016641 | oxidoreductase activity, acting on the CH-NH2 group of donors, oxygen as acceptor | 0.004101 | 4 |
